# Identifying Gene Set Association Enrichment Using the Coefficient of Intrinsic Dependence

**DOI:** 10.1371/journal.pone.0058851

**Published:** 2013-03-14

**Authors:** Chen-An Tsai, Li-Yu Daisy Liu

**Affiliations:** Department of Agronomy, Biometrics Division, National Taiwan University, Taipei, Taiwan; Memorial Sloan Kettering Cancer Center, United States of America

## Abstract

Gene set testing problem has become the focus of microarray data analysis. A gene set is a group of genes that are defined by a priori biological knowledge. Several statistical methods have been proposed to determine whether functional gene sets express differentially (enrichment and/or deletion) in variations of phenotypes. However, little attention has been given to analyzing the dependence structure among gene sets. In this study, we have proposed a novel statistical method of gene set association analysis to identify significantly associated gene sets using the coefficient of intrinsic dependence. The simulation studies show that the proposed method outperforms the conventional methods to detect general forms of association in terms of control of type I error and power. The correlation of intrinsic dependence has been applied to a breast cancer microarray dataset to quantify the un-supervised relationship between two sets of genes in the tumor and non-tumor samples. It was observed that the existence of gene-set association differed across various clinical cohorts. In addition, a supervised learning was employed to illustrate how gene sets, in signaling transduction pathways or subnetworks regulated by a set of transcription factors, can be discovered using microarray data. In conclusion, the coefficient of intrinsic dependence provides a powerful tool for detecting general types of association. Hence, it can be useful to associate gene sets using microarray expression data. Through connecting relevant gene sets, our approach has the potential to reveal underlying associations by drawing a statistically relevant network in a given population, and it can also be used to complement the conventional gene set analysis.

## Introduction

The interactions of genes usually take place in the signaling pathways, networks, or other biological systems. In particular, the interactions between or among multi-dimensional gene sets in a given biological system have been demonstrated in a functional network [1], [2], [3], [4], [5], [6]. By taking advantage of high throughput data and many fine algorithms, we have the opportunity to predict many novel interactions among gene sets, which may resolve the complexity in health and disease biology system-wide. A set of genes with related functions can be grouped together and referred to as a ‘gene set’. The gene sets (possibly overlapped) are usually defined by functional categories or metabolic/signaling pathways, and annotation resources for gene sets can be found in several publicly available annotation databases such as the Kyoto Encyclopedia of Genes and Genomes (KEGG) [7], Biocarta (http://www.biocarta.com/), Gene Ontology (GO) [8], and GenMAPP [9], [10]. If the expression levels of a gene set are significantly associated with the clinical outcomes/phenotypes, then we can say that this gene set is ‘differentially expressed’. Many statistical approaches, such as gene set enrichment analysis (GSEA) methods [7], [8], are used to determine whether functional gene sets express differentially (enrichment and/or deletion) in variations of phenotypes. Readers are referred to [9] for the review of current GSEA algorithms.

In this study, we deal with the gene sets in a different way. Instead of identifying differentially expressed gene sets, we aim to exploit the dependence structure among gene sets and propose a testing strategy for identifying gene set pairs with statistically significant coherence by using microarray data. We refer to this approach as ‘Gene Set Association Analysis’ (GSAA) to distinguish it from GSEA methods. More specifically, our approach provides a statistical framework for analyzing coherence of expression profiles in gene sets, which measure functional module co-regulation. Most biological systems are composed of complex interactions of functional gene modules. In an attempt to understand the co-expression networks, GSAA is used to study whether gene sets with common functionality show high degrees of co-expression or whether two gene sets show significantly correlated expression in tumor cells but weakly correlated expression in normal cells. Such coherent or incoherent correlations between gene sets may indicate different types of gene set interactions which play an important role in complex diseases. Although the associations between two individual genes have been explored in depth, to the best of our knowledge, only little attention has been given to analyzing the association between two gene sets. One reason may be that the statistical measures are to pick up the most relevant associations, which are in consensus in a given population, while most of the associations are chaotic and only some of them are in consensus. Another reason might be the lack of appropriate statistical measures for two multi-dimensional variables. The canonical correlation (see, e.g., [10]) and the projection pursuit regression [11] are two conventional methods for evaluating the association between two multi-dimensional variables. However, they have several limitations. The canonical correlation assumes normality, which is often violated in real experimental data. Besides, the canonical correlation adopts Galton-Pearson's correlation coefficient, which is designed to capture only linear relationships. The projection pursuit regression considers more general forms of associations, but it would put too much emphasis on numerous smoothing processes even though the smoothing results of irrelevant ones might be disregarded in the end.

To develop a statistical measure describing the general dependence between two gene sets, it is reasonable to start with the definition of independence in statistical theory. Conceptually, when two gene sets are not related, the expressions of one gene set provide little information about predicting the expressions of the other gene set. That means the distribution of the expression levels for the target gene set would not be altered much even though additional information of another independent explanatory gene set is provided. The pattern of the expressions for the target gene set alone and that of the expressions for the target gene set, given the explanatory gene set, are referred to as the marginal and the conditional distributions, respectively. If two gene sets are independent of each other, one can expect the marginal and the conditional distributions would be very similar to each other. Therefore, the dissimilarity between the marginal and conditional distributions can serve as a measure of association between two gene sets – a larger dissimilarity implies a higher association. This type of measure requires neither distributional (e.g. normal) nor functional (e.g. linear) assumptions on the observations, and it may possibly obtain a wider range of associations between two gene sets than the regression-based measures.

There already exist some statistics to measure the discrepancy between two distributions, including the Kolmogorov-Smirnov statistic [12], the Cramér von-Mises statistic [13], the Kullback-Leibler distance [14], and the Hellinger distance [15]. Among these conventional methods, the coefficient of intrinsic dependence, or CID, has been recently proposed [16], [17]. The CID takes any real value between 0 and +1 inclusive. It is +1 in the case of full dependence and is 0 in the case of independence. As the level of dependence ascends, the CID value goes from 0 to 1. Our previous work has demonstrated that the CID, as a univariate measure of association, was capable of identifying essential features [18], [19]. By definition, the CID is also applicable in multivariate cases. In this paper, we aim to detect the association among sets of genes using the extension of the CID. It was shown from the simulations that the CID outperformed the conventional methods to detect associations in general forms in terms of control of type I error rate and power. We further conducted GSAA using the CID on the microarray expression datasets in the breast cancer samples. The results showed that the associations between gene sets changed across different clinical cohorts when using an unsupervised learning. In the examples of the supervised GSAA, the CID was utilized to predict the co-expressed TF(s) and cofactor(s) that possibly form cistromes [20] which regulate a gene set coding for a signature and a pathway, respectively. Therefore, we concluded that the CID is an appropriate statistic which allows one to assess the underlying system-wise nonlinear association between two gene sets.

## Materials and Methods

In this section, we describe the statistical measures mentioned in this study and the simulation settings, as well as the availability of real microarray datasets. Throughout this section, we denote the predictor gene set with *p* genes and the target gene set with *q* genes as ***X*** and ***Y***, respectively (*p*, *q*≥1). Each gene set has *N* realizations. More specifically, let (***x*** *_i_*, ***y*** *_i_*) be the *i*th paired observation of (*X*, *Y*), where *i* = 1, …, *N*, ***x*** *_i_* = (*x_i_*_1_, *x_i_*_2_, …, *x_ip_*) and ***y*** *_i_* = (*y_i_*_1_, *y_i_*_2_, …, *y_iq_*).

### The Coefficient of Intrinsic Dependence (CID)

The CID value of ***Y*** given ***X*** is defined as follows:



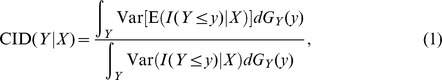
 where ***G_Y_***(.) is the marginal cumulative distribution function (cdf) of ***Y***, and *I*(*A*) is an indicator function such that



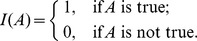



It has been shown that the CID quantifies the discrepancies between the marginal and conditional cdfs of ***Y*** [17]. When ***X*** and ***Y*** are nearly independent, the knowledge of ***X*** provides little information about ***Y***. The conditional and marginal distributions of ***Y*** are therefore similar to each other, which makes the numerator of the CID nearly 0. On the other hand, if two variables are highly relevant, one can easily discriminate the object only by using the knowledge of ***X***. In these cases, the CID yields values close to 1.

The estimation of the CID is demonstrated using a toy example shown in Table 1. In the example, there are five realizations (named r1 to r5) for p = 3 predictor variables and q = 2 target variables. First, the CID promotes subgrouping the sample of size *N* into *K* subgroups by hierarchical clustering based on Euclidean distance according to the observed values of ***X***s (Table 1). The options of subgrouping will be described later in the ‘Subgrouping strategy’ section. In the toy example, (r4, r5) are closest to each other (Euclidean distance = 0.3976) and (r1, r2) are second closest (Euclidean distance = 0.8956). If letting the number of subgroups *K* = 3, the five realizations are subject to three subgroups named (r1, r2)-group, r3-group, and (r4, r5)-group, respectively. In each subgroup *s* (*s* = 1, …, *K*), the following quantity was evaluated:

**Table 1:** Toy example of the CID calculation.

		Realizations				
		r1	r2	r3	r4	r5
Predictor	X1	−0.38	−0.24	−0.32	−0.05	0.05
	X2	0.27	0.36	−0.36	0.25	0.09
	X3	1.82	0.94	−0.62	0.37	0.02
Target	Y1	0.17	4.33	−0.87	−2.37	2.55
	Y2	1.88	1.83	0.61	0.43	2.03
Distance	r2	0.8956				
	r3	2.5207	1.7200			
	r4	1.4872	0.6108	1.1938		
	r5	1.8594	1.0017	0.8654	0.3976	
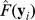		0.6	0.6	0.4	0.2	0.8
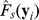	(r1, r2)-group	0.5	0.5	0	0	0.5
	r3-group	1	1	1	0	1
	(r4, r5)-group	0.5	0.5	0.5	0.5	1

The data consisted of the 5×2 target and the 5×3 predictor. The Euclidean distances between any two realizations, the estimations of the marginal distribution, 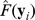, and conditional distributions, 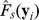
’s, were also shown.



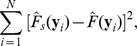
 where



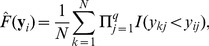
(2)




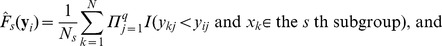
(3)




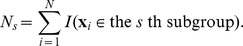




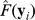
 is the estimation of the marginal distribution of Y given the realization, *y_i_*. For example, given *y_i_* = r1 in the toy example:



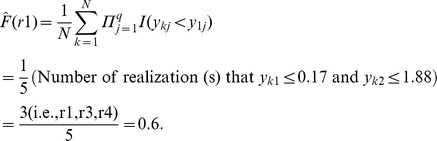



Similarly, 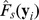 is the estimation of the conditional distribution of Y obtained by only comparing the observations within the *s*th subgroup to the given realization. Within the (r4, r5)-group, for example, the conditional distribution of Y given the realization r1 is



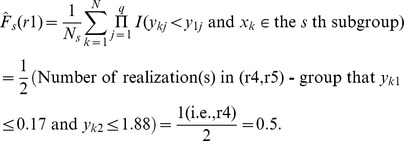



The estimations of the marginal and conditional distributions given all realizations are listed in Table 1. A weighted average is taken to account for all discrepancies measured within different subgroups at the *i*th realization,



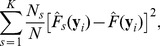
(4)


The estimate of the CID is



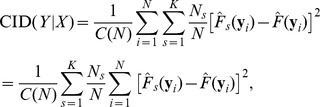



where *C*(*N*) is a denominator that ensures the CID values are within the range [0,1]. More specifically,



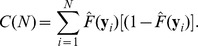



In the toy example, 
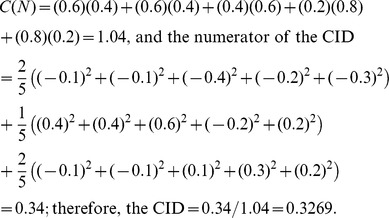



We note that the CID is asymmetric, meaning the CID(***Y***|***X***) is not necessarily equal to the CID(***X***|***Y***). The asymmetry of the CID may reflect uneven levels of influence of one variable (gene set) on another. If a symmetric measure is desired, one can simply take the average of the CID(***Y***|***X***) and CID(***X***|***Y***) as the level of dependence between ***X*** and ***Y***.

### Regularized canonical variates for high-dimensional gene set data

To reduce the computation complexity and to retain the common dominant pattern within gene sets, we consider the first few (i.e., 2 or 3) pairs of canonical variates for the CID estimation for high-dimensional gene set data (say, *p*≥10 or *q*≥10). Once the first few pairs of canonical variates are determined, they can be used for estimation of the CID. However, when the number of genes in the gene set is greater than the number of samples, or genes within a gene set are highly correlated, the sample covariance matrix is singular and ill-conditioned. In this article, we propose a dimensional reduction method for estimation of the CID that is based on the regularized canonical analysis of gene set data. The regularized canonical variates proposed by Leurgans et al. [21] are used to deal with this problem via a regularization procedure. Consider two gene set expression matrices ***X*** and ***Y*** of dimensions 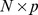 and 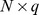 respectively with the column corresponding to standardized gene expression values (mean 0 and variance 1). We denote by 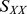 and 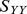 the sample covariance matrices for gene sets ***X*** and ***Y*** respectively, and by 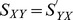 the sample cross-covariance matrix between ***X*** and ***Y***. The *k*th pair of canonical variates is defined as the linear combinations of columns 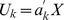 and 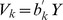 having unit variances which maximize the correlation among all choices ***a*** *_k_* and ***b*** *_k_* uncorrelated with the previous *k*-1 pairs of canonical variates. Without loss of generality, we assume that 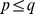 and 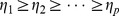 are eigenvalues of 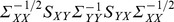 in decreasing order, where the regularized covariance matrices are defined as 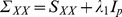 and 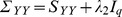. Then, the pair of coefficient vectors of ***a*** *_k_* and ***b*** *_k_* can be estimated by 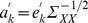 and 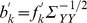, respectively, where the vector ***e*** *_k_* is the eigenvector corresponding to the eigenvalue 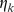 of 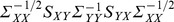 and the vector ***f*** *_k_* is the eigenvector corresponding to the eigenvalue 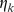 of 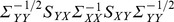. The regularization parameters can be chosen to maximize the correlation of the first pair of canonical variates via the leave-one-out cross-validation suggested in [21].

### Conventional methods of association for comparison

We compared the CID with two types of conventional measures of associations, the regression-based methods and the distribution-based methods. The regression-based methods included the canonical correlation (see, e.g., [10]) and the projection pursuit regression [11]. They were abbreviated as CanCor and PPR in context. Both CanCor and PPR define association between ***X*** and ***Y*** using a general form



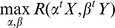



where *α^t^* ***X***, *β^t^* ***Y*** are linear combinations of the original variables, and *R* is a univariate association measure. The CanCor takes *R* for the Galton-Pearson correlation coefficient and the PPR takes *R* for the correctness of prediction by nonparametric regression such as Friedman's super smoother or the smoothing spline. The *stat* package in freely-accessible software R [22] provides two functions, cancor and ppr, to perform CanCor and PPR. To compare with the CID, we recorded the largest correlation retrieved from the output of cancor and the residual sum of squares from the output of ppr. It is intuitive that a larger correlation for CanCor or a smaller residual sum of squares for PPR implies a higher level of association.

Two distribution-based methods were considered in this study. They were the Kullback-Leibler distance [14] and the Hellinger distance [15] (abbreviated as KLD and HD, respectively, in context). The rationale of distribution-based methods for GSAA is that if the predictor and the target gene sets are independent of each other, one can expect that the marginal and the conditional distributions of the target gene sets would be very similar to each other. Let *f_X_* and *f_Y_* be the marginal probability density functions (pdfs) of *X* and *Y*, respectively (*p*, *q*≥1). Also let *f_X,Y_* be the joint pdf of *X* and *Y*. Then the conditional pdf of *Y* given *X* could be present as *f* ***_X_***_|***Y***_ = *f* ***_X_***_*,****Y***_ / *f* ***_Y_***. The Kullback-Leibler distance (KLD) and the Hellinger distance (HD) were adopted to measure the dissimilarity between the marginal and conditional distributions of the target gene expressions. Let


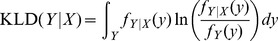
, and 
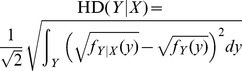
. Given *N* realizations, the conditional and marginal pdfs of *Y* can be estimated as follows: Each dimension of *Y* in the sample was discretized into *r* = 3 subgroups by its sample quantiles. Then the marginal pdf of *Y* was estimated by



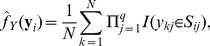
 where *S_ij_* represented the subgroup that *y_ij_* belonged to. To estimate the conditional pdf *f_Y|X_*, we first divided the sample into *K* subgroups by hierarchical clustering according to the observed values of *X*. Then the conditional pdf of *Y* given the *s*th subgroup of *X* was estimated by



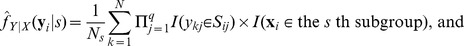





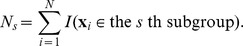



The estimates of KLD and HD were formulated as 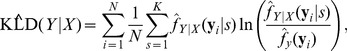
and 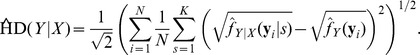



### Subgrouping strategy

By definition, the distribution-based methods (CID, KLD and HD) measure the discrepancy between marginal and conditional distributions. The estimate of distribution from the sample is called the empirical distribution. In particular, histogram-like methods using subgrouping are widely adopted in estimating empirical distributions [23]. One way of subgrouping is to categorize the *d*th dimension of *X* into *r_d_* subgroups by its sample quantiles. A combination of *p* dimensions discretizes the sample into 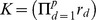 subgroups. To equally weight all dimensions of *X*, they usually set *r_d_* = *r* for all *d* and *K* = *r^p^*. The quantile method considers that each subspace is equally important throughout the range of each dimension of *X* and is expected to yield an unbiased estimate of discrepancies. However, it faces the curse of dimensionality when *p* increases [24]. That is, the observations distribute sparsely and most of the combinations of the subgroups have zero or too few observations. The quantile method has another technical problem – the production of *r_d_* may not be the desired number of subgroups. For example, when *p*  = 3 and *r_d_* = 2, *K* = 8; when *p* = 3 and *r_d_* = 3, *K* = 27; it is not possible to divide the sample into *K* = 10 subgroups when *p* = 3 for any *r_d_*.

**Figure 1:**
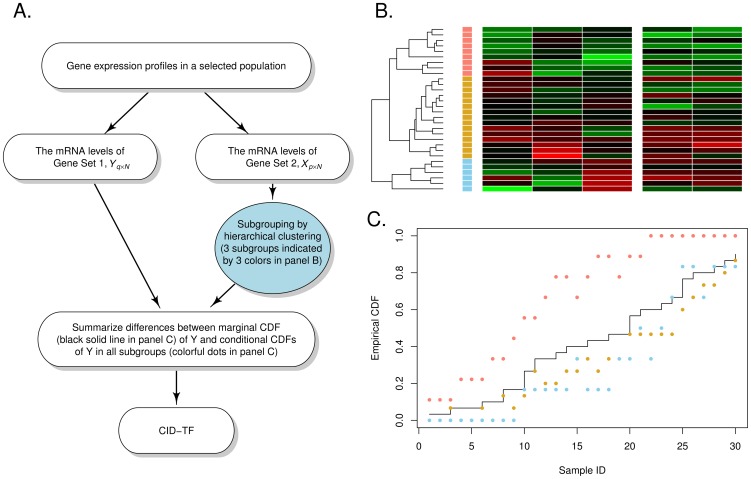
Flow chart of GSAA based on the CID and hierarchical clustering.

In this research, we propose to partition the sample by a hierarchical clustering, while the CID algorithm remains robust to other clustering methods (e.g., kmeans [25] or SOM [26]; see Figure S1). Hierarchical clustering is a commonly used algorithm for dividing the sample into more homogeneous subgroups. Our intention of subgrouping by hierarchical clustering aimed to mimic biological systems in which similar expression pattern may reflect the similar biological event shared by the members within a subgroup. In this study, the subgrouping preceded as follows (see also Figure 1): First, a hierarchical clustering algorithm with complete linkage based on the Euclidean distances between *x_i_*'s was performed. A tree-shape diagram, or dendrogram, was then used to further cluster the sample into the desired number of subgroups. Figure 1B showed one example of dividing 30 realizations of a variable (gene set) with 3 dimensions (genes) into three subgroups (marked blue, brown, and pink, respectively) by hierarchical clustering. This method can be applied to any number of subgroups. However, the sizes of the subgroups may be extremely unbalanced. For example, in Figure 1B, the sizes of the subgroups were 6, 15, and 9, respectively.

### Assessment of the significance by random permutations

The null distribution of all association measures under independence was generated by random permutations. For the CID, we re-computed the CID value given the random permuted labels of subgroups. For CanCor, PPR, KLD and HD, the estimates were recorded respectively after randomly permuting the rows (observations) of *X*. Random permutation was repeated 1,000 times and yielded 1,000 internal control values for each measure under independence. Let *E_0_* be the estimate of an association measure from the sample, and *E_i_* be the estimate for that measure from the *i*th random permutation. The permuted p-value for each association relationship between two variables of interest was determined by 
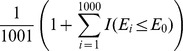
 for PPR, or 
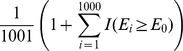
for the other methods, where



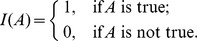



In this research, we claim that *X* and *Y* are significantly dependent if the permuted p-value was less than or equal to the nominal level of α.

### Simulation methods

We evaluated the performance of our proposed method and compared it with four conventional methods in terms of control of type I error and power using the Monte Carlo simulation. Two models were used to simulate correlated gene sets data: the multivariate normal model and the non-linear model. The multivariate normal model was formulated as



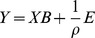
(5)


where 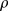
was a constant, *Y* was a *N* by *q* observed matrix of *q* response variables on each of *N* objects in the sample, *X* was a *N* by *p* matrix describing the observed values of explanatory variables *X*, *B* was a matrix of regression parameters, and *E* was a matrix of unobserved random errors whose rows for given *X* were uncorrelated, each with mean 0 and common covariance matrix Σ,



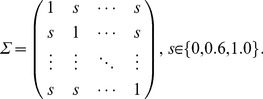
(6)


Here, we considered a common intra-gene set correlation structure with covariance *s*. The covariance *s* was set at 0, 0.6 and 1.0. To simplify the scenario, we let *B* be the matrix with all elements set to 1. To model an association between a pair of gene sets, we allowed the strength of dependence between *X* and *Y* to vary with the inter-gene set correlation 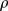. In this study, the correlation 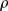 was set at 0.2, 0.4, 0.6, 0.8 and 1.0. We also considered a null model with 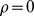 (independent model) to assess the type I error rate.

The nonlinear model was motivated by the Friedman model [27]. Suppose *X* = (*X*_1_, *X*_2_, …, *X*_6_)*^T^* where *X_i_*s were distributed independently as Uniform(0,1) and *Y* = (*Y*_1_,*Y*_2_) was determined by the following equation:



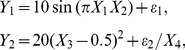
(7)


where *ε*_1_ and *ε*_2_ were random numbers distributed as Normal with mean = 0 and standard deviation = 1. In the model (7), (*X*_1_, *X*_2_) and (*X*_3_, *X*_4_) were dependent on *Y* in two ways – the values of (*X*_1_, *X*_2_) would change the means of *Y*_1_ nonlinearly; the values of (*X*_3_, *X*_4_) would alter the degrees of variation of *Y*_2_. However, (*X*_5_, *X*_6_) was independent of both *Y*_1_ and *Y*_2_. The CID values of *Y* given (*X*_1_, *X*_2_), (*X*_3_, *X*_4_) and (*X*_5_, *X*_6_) were computed respectively, to see the capability of the statistical measures to identify different forms of association.

For each of the models (5) and (7), the simulation data were replicated 100 times with a sample size of 100. The p-values were based on 1000 permutations. Power was then estimated as the proportion of significance using the nominal level of 0.05. To estimate the CID, we set the number of subgroups to 5 in each simulation.

### Microarray expression data

All clinical data arrays used in this study were from a patient cohort (from 2002 to 2005) collected at National Taiwan University Hospital (NTUH). These arrays were generated using the Human 1A (version 2) oligonucleotide microarray from Agilent Technologies, according to the methods provided by the manufacturer. The expression dataset can be downloaded from the GEO database (Accession numbers GSE24124, GSE17040 and GSE9309). The dataset includes gene expression of the tumor tissues from 181 patients as well as the gene expression from the adjacent non-tumor tissues of 25 patients (Table S1). Microarray raw data went through data processing which included background correction, elimination of poor quality spots, and log transformation of RNA measures relative to a reference (Stratagene's human common reference RNA) using a base-2 logarithm. The average of the expression levels and of the feature numbers of replicated probes were then taken before statistical analysis; the average feature numbers were initialized with a capital letter ‘C’ to distinguish them from the original feature numbers.

### Unsupervised GSAA on KEGG and BioCarta gene sets

The CID was further exercised via unsupervised learning to identify the sets of genes that are associated with (or possibly regulated by) a target gene set. A total of 186 gene sets from KEGG [28] and 217 gene sets from BioCarta (http://www.biocarta.com/) were downloaded from the GSEA website (http://www.broadinstitute.org/gsea/index.jsp). We analyzed the expression of 25 paired tumor and non-tumor samples for the unsupervised GSAA (Table S1). The 25 tumor samples and the 25 non-tumor samples were designated as 25T and 25N, respectively. Only probes with no missing value in the microarray expression dataset were considered. The online converting tool DAVID [29], [30] was used to map the Entrez ID of the genes obtained from the GSEA website to the Agilent probe ID (Table S2).

In the context of microarray experiments, the number of genes in a gene set may be greater than the number of samples. In this case, the value of the CID may not reflect the degree of dependence due to the discreteness of the empirical distribution function when the sample size is relatively small compared with the dimension. Therefore, we selected up to the first three pairs of regularized canonical variates for assessing statistical significance of the latent correlation between the pair of gene sets. There are two CID values for every combination of two gene sets --- the first CID value comes from that one gene set is set to be the target while the other is set to be the predictor, and the second CID value from that the target and predictor gene sets are swapped. Therefore, a total of 17,205 and 23,436 gene pairs in KEGG and Biocarta datasets were inspected, and the analyses resulted in 34,410 and 46,872 CID values, respectively. In the unsupervised gene set analysis, the number of subgroups was set to 3 when estimating the CID.

Different approaches other than random permutations were adopted to assess the significance of gene set association more efficiently. Suppose there are *G* gene sets in the database (*G* is equal to 186 in the KEGG database and 217 in the BioCarta database). Let the *i*th gene set *g_i_* (*i* = 1, …, *G*) be the target (i.e. *Y* in Equation (1)). The other *G* – 1 gene sets were set in turns to be the predictor (i.e. *X* in Equation (1)) and yielded accordingly *G* – 1 CID values. Given a predictor gene set *g_j_* (*j* = 1, …, *G* and *j*≠*i*) we computed the adjusted values of the CID by



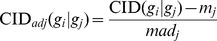
 where *m_j_* and *mad_j_* was the median and median absolute deviation (MAD) of CID(*g*_1_|*g_j_*), …, CID(*g_R_*|*g_j_*), respectively. The adjusted values of the CID greater than 3.5 were potential outliers [31] and were declared to be significant in this research.

### Supervised GSAA to select the signaling transduction pathways relative to the set of transcriptional regulators in a population

**Figure 2:**
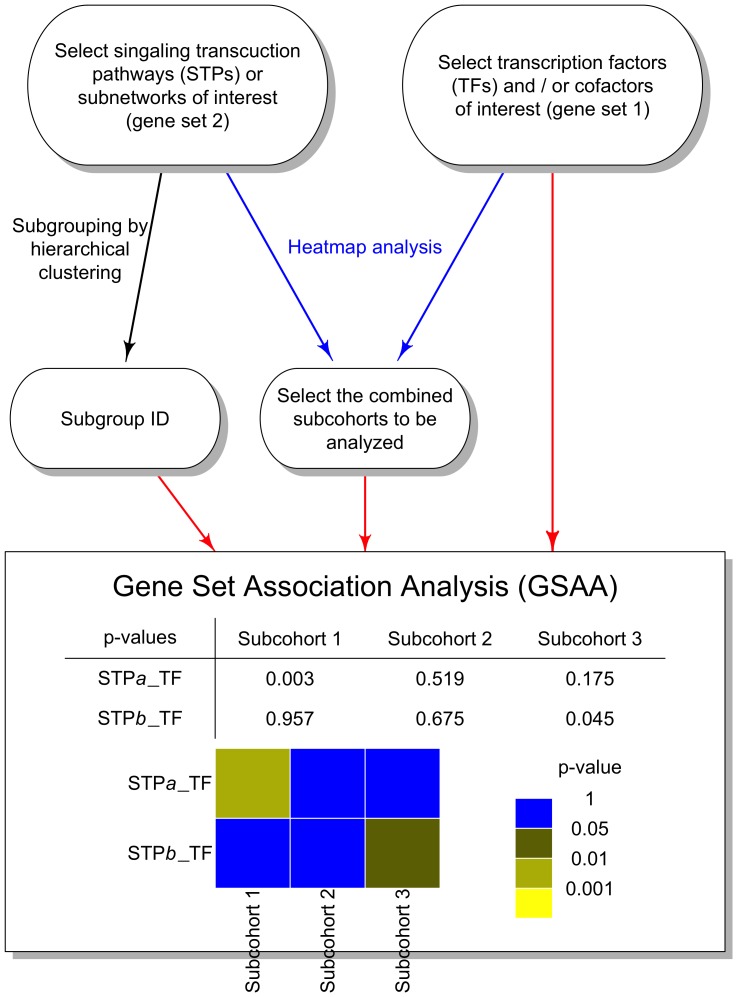
Flow chart for running a supervised GSAA in a selected cohort. Three major steps are included as follows. First, one has to select a gene set containing transcription factors and/or cofactors of interest in this study. Another gene set is selected from the selected feature (signaling transduction pathway (STP) or subnetwork (SNET)) of interest. Second, a hierarchical clustering is made to divide the similar gene expression patterns of gene components in the STP or in the SNET into subgroups. Third, if the co-existing event is true, the *p*-value for GSAA would be small. A color scale bar represents the color gradient for a series of *p*-values from GSAA to assist in the visualization of GSAA results when they are presented in a diagram.

Different patterns of gene set associations may provide an insight into the analysis of transcriptional regulation. In the supervised GSAA, the transcription factors of interest were designated as gene set 1, and the genes in the selected signal transduction pathways were designated as gene set 2. Note that the signal transduction pathway is normally only partially regulated by the TFs. This could reduce the sensitivity of a bottom-up approach. However, this bottom up approach may provide an instant biological insight in a semi-blind experiment, before running a more sensitive top-down analysis. Figure 2 provides a flow chart representing the general steps of performing analysis of the association between the signaling transduction pathways and the set of transcriptional regulators in a given population in this study. We first designed the cohorts of interest with their counterparts. The analyses were then performed on the microarray data from each cohort with their counterparts. The cohorts under study and their counterparts for the supervised GSAA were listed in Table S3. In each cohort, only the arrays containing no missing values for all genes in gene sets 1 and 2 would be adopted for further use. In the supervised gene set analysis, the number of subgroups was set to approximately one-tenth of the cohort size when estimating the CID. The *p*-values for the CID estimates for our supervised GSAA were determined through the same random permutations described in the ‘Assessment of the significance by random permutations’ section. We claimed that the TFs were significantly associated with the genes in the signal transduction pathway if the *p*-value ≤ 0.05.

In the first example of supervised GSAA, gene set 1 contained two transcription factors (TFs), *STAT3* and *MYC*. The genes in two signal transduction pathways (STPs), proteasomes (containing 43 genes) and PDGFRB (containing 65 genes), were in turn set to be gene set 2. Table S4 provided the complete list of genes in the two STPs. We designed two tumor cohorts with their counterparts (Table S3) based on two commonly used clinical pathological indices, ER and HER. The two tumor cohorts were designated as LumA (ER(+) and HER(−)) and LumB (ER(+) and HER(+)), respectively. The counterpart of the tumor cohorts were 18 arrays from the adjacent non-tumor tissues collected from the ER+ patients. Therefore, the cohort sizes were 60 and 48, and numbers of subgroups were 6 and 5 for LumA and LumB, respectively. In the second example of supervised GSAA, gene set 1 contained transcription factors of interest and gene set 2 contained the target genes in the subnetwork regulated by the transcription factors (Table 2) [19]. The designed cohort (152A) consisted of 61 Group IE (ER(+), PR(+)) arrays and 91 arrays from ER(−) patients (Table S3). The number of subgroups was set to 15 in this example.

**Table 2:** List of transcription factors and target genes in the second example of supervised GSAA.

Name of TF subnetwork	TFs (gene set 1)	Subnetwork target genes (gene set 2)
EE1a	ESR1(5561)	ACTR10(17602)
	E2F1(7852)	ADAMTS5(11603)
		AGGF1(9464)
		AGGF1(10316)
		BUB3(19468)
		CD44(14592)
		CNOT4(6977)
		SFRS1(16354)
EE1b	ESR1(5561)	ALG8(14180)
	E2F1(7852)	ATAD2(C11227.3)
		CD44(14592)
		DTL(C10948.8)
		IVNS1ABP(11136)
		RACGAP1(2299)
		RFC3(18703)
		YBX1(4742)
EG	ESR1(5561)	CCT5(815)
	GATA3(14967)	CPSF2(15856)
		DHFR(18343)
		GART(11131)
		KPNB1(9017)
EGE1	ESR1(5561)	KIF2C(19023)
	GATA3(14967)	CDCA8(9984)
	E2F1(7852)	

Four subnetworks have been analyzed. The transcription factors (TFs) of interest were set as gene set 1 and the target genes in the subnetwork regulated by the transcription factors were set as gene set 2.

## Results

### Simulation results

In the simulation study, we explore the performance of our proposed method in identifying the enriched correlation between two gene sets through observing their mRNA expression levels. Let the *p* by *N* matrix *X* be the expression levels of a set of *p* genes and the *q* by *N* matrix *Y* be the expression levels of another set of *q* genes, where *N* is the sample size. The goal of GSAA is to quantify the dependence between *X* and *Y* using a single number. Five statistical measures were evaluated for this purpose through the simulation study. They were the coefficient of intrinsic dependence (CID), the canonical correlation (CanCor), the projection pursuit regression (PPR), the Kullback-Leibler distance (KLD) and the Hellinger distance (HD). CanCor and PPR were classified as regression-based measures because regression analysis is involved in both methods. Furthermore, CID, KLD and HD were classified as distribution-based statistics because they account for discrepancy between marginal and conditional distributions (see the ‘Materials and Methods’ section).

**Figure 3:**
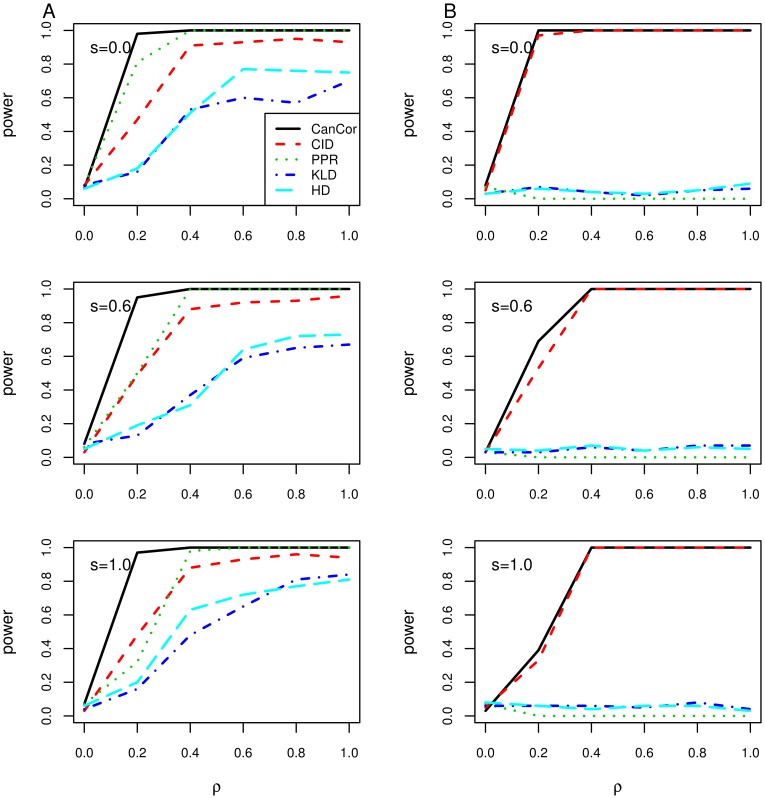
Power analysis of five methods in the multivariate normal model at level α = 0.05. (A) True positive rate under different levels of association for p = 5 and q = 2. (B) True positive rate under different levels of association for p = q = 30. From top to bottom panels, the intra-gene set correlation coefficients were *s* = 0, 0.6, and 1, respectively.

Two experimental designs were simulated according to the multivariate normal model (5). The first design was an experiment with small-size gene sets with *p* = 5 and *q* = 2. In the second design we considered a larger-size gene sets, each with 30 genes (*p* = *q* = 30). We compared the performance of these statistical methods to identify different levels of linear association between the *p*-dimensional predictor variable *X* and the *q*-dimensional target variable *Y* in terms of type I error rate and power. The model used the constant 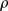 to represent the level of association and the constant *s* to represent the possible dependency within *Y* (Equations (5) and (6) in the ‘Materials and Methods’ section). For each combination of 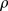 and *s*, 100 replications were performed for each statistical measure with a sample size of 100. Table 3 showed the empirical type I errors using the nominal levels of 0.01 and 0.05 for each scenario. The type I errors from the CID and CanCor were reasonably close to or below the nominal level. PPR appeared to have an inflated type I error rate in most cases. CanCor, KLD and HD showed slight anti-conservatism in the case of small-size gene sets when *p* = 5 and *q* = 2. Next, we compared the power of the CID with the other four approaches to detect a significant association. Figure 3 illustrated the empirical powers using the nominal level of 0.05 for 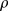 = 0.2, 0.4, 0.6, 0.8 and 1.0. As expected, CanCor had a greater power if the data was normally distributed, especially for smaller intra-gene set correlation (s≤0.6). In the case of small-size gene set, PPR performed slightly better than the CID while PPR was unable to adequately control the type I error rate. The other two distribution-based methods, KLD and HD, had the least power in all cases in the multivariate normal model (Figure 3A). The power of all methods increased gradually with increasing inter-gene set correlation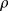. On the other hand, the power of the CID and CanCor was comparable and both outperformed other approaches when the gene set size was modest with *p* = *q* = 30 (Figure 3B).

**Table 3:** Type I errors of five methods for the linear model at levels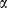 =  0.01 and 0.05.

Gene set sizes	Intra-Correlation (s)	Nominal levels	Methods				
			CID	CanCor	PPR	KLD	HD
p = 5;q = 2	s = 0.0	0.01	<0.01	0.01	0.02	0.01	0.01
		0.05	0.08	0.06	0.08	0.08	0.06
	s = 0.6	0.01	<0.01	0.03	0.02	0.05	0.01
		0.05	0.03	0.08	0.06	0.08	0.05
	s = 1.0	0.01	<0.01	<0.01	<0.01	<0.01	0.01
		0.05	0.03	0.07	0.07	0.04	0.06
p = q = 30	s = 0.0	0.01	0.02	0.03	0.01	0.02	0.02
		0.05	0.05	0.08	0.07	0.03	0.03
	s = 0.6	0.01	<0.01	0.01	<0.01	<0.01	0.01
		0.05	0.03	0.03	0.03	0.03	0.05
	s = 1.0	0.01	0.01	0.02	0.02	<0.01	<0.01
		0.05	0.05	0.03	0.07	0.06	0.08

**Figure 4:**
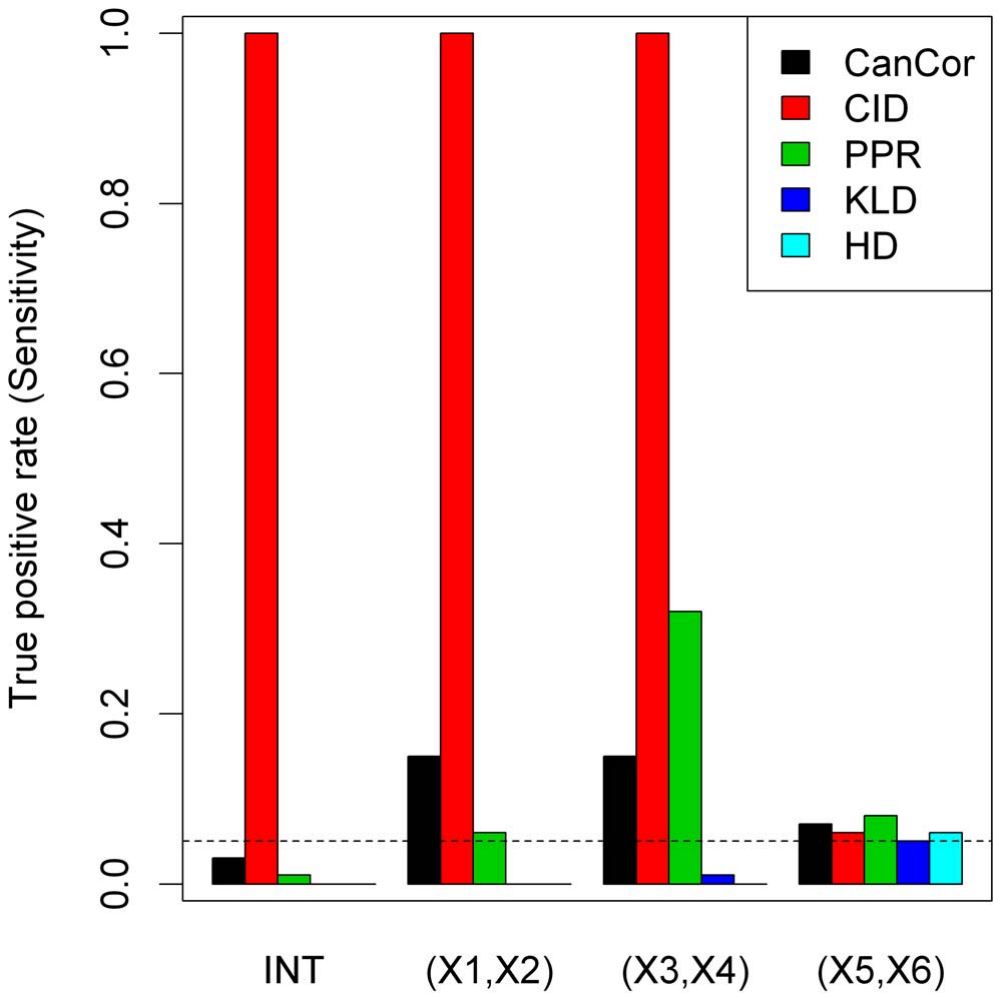
Comparing the performance of five methods in the nonlinear model. The height of the bars shows the true positive rate under different levels of association. The dashed line showed the nominal level of 0.05 for detecting 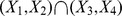 (denoted as ‘INT’), (*X*_1_, *X*_2_), and (*X*_3_, *X*_4_).

To explore the robustness of our proposed method with regard to non-linear association data, we simulated non-linearly associated gene set data according to model (7). Figure 4 showed the average power over 100 simulations for each method using the nominal level of 0.05. Under the null hypothesis, the type I errors of all methods were close to the nominal level of 0.05 (0.07 for CanCor, 0.06 for CID, 0.08 for PPR, 0.06 for KLD, and 0.05 for HD) when testing on (*X*_5_, *X*_6_). Under the alternative hypothesis of association, the CID appeared to be the most powerful method in detecting the non-linear association in either (*X*_1_, *X*_2_) or (*X*_3_, *X*_4_) (both had power equal to 1), whereas CanCor and PPR had power less than 0.4. If we considered the scenario to detect both (*X*_1_, *X*_2_) and (*X*_3_, *X*_4_) at the same time (denoted ‘INT’ in Figure 4), the power of the CID was also equal to 1; whereas PPR and CanCor had power 0.03 and 0.01, respectively. Both KLD and HD had poor performance in detecting non-linear associations; the true positive rates were all close to 0 regardless of detecting (*X*_1_, *X*_2_), (*X*_3_, *X*_4_), or their intersection. As a result, the CID provided a more powerful test than the other methods to detect the non-linear association between gene sets.

### Unsupervised gene set association analysis using the CID

**Figure 5:**
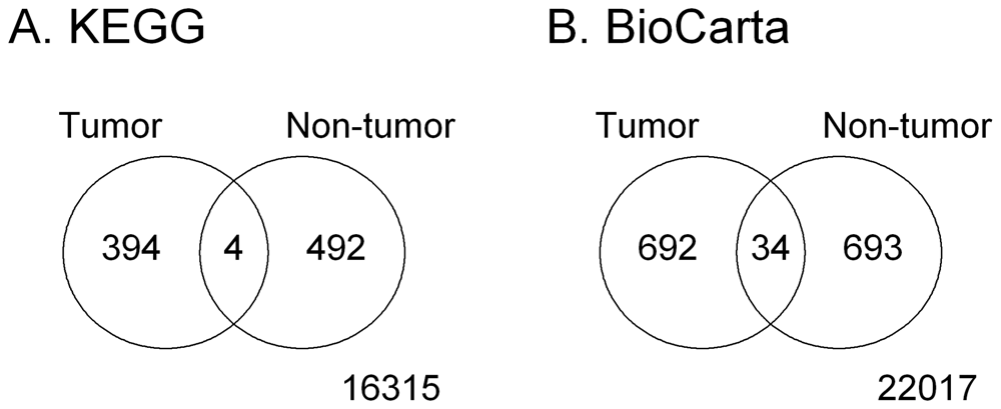
Venn diagrams for the number of significant gene-set pairs in analyses. (A) KEGG database; (B) BioCarta database.

The CID was used to further identify associated gene sets by using microarray expression data. The microarray expression data consists of 50 samples, 25 from tumor samples and 25 from non-tumor samples; they were designated as 25T and 25N, respectively. The pairwise associations of the gene sets in KEGG [32] and BioCarta (http://www.biocarta.com) were analyzed for 25T and 25N and the numbers of significantly associated gene-set pairs were shown in Figure 5. We observed that the significant gene-set pairs were very different in tumor and non-tumor samples. There were 890 out of 17,205 (5.17%) pairs of gene sets declared significant in the KEGG database; 0.45% (4 out of 890) were significant in both 25T and 25N, 44.27% (394 out of 890) were significant only in 25T, and 55.28% (492 out of 890) were significant only in 25N. In the BioCarta database, we examined 23,436 pairs of gene sets and 1,419 (6.05%) of them were significant; 2.40% (34 out of 1,419) were significant in both 25T and 25N, 48.77% (692 out of 1,419) were significant only in 25T, and 48.83% (693 out of 1,419) were significant only in 25N. The result implied that the relation between gene sets might be altered along with the development of breast cancer.

Tables S5 and S6 presented the pairs of gene sets that were significantly related in the KEGG and BioCarta databases, respectively. Columns corresponded to the target gene sets, *X*, and rows to the predictor gene sets, *Y*. To emphasize the outcomes in different cohorts, significantly associated gene-set pairs in the tumor sample were labeled in red; significantly associated gene-set pairs in the non-tumor sample were labeled in green, and significantly associated gene-set pairs in both samples were labeled in yellow in Tables S5 and S6. In the KEGG database, there were 154 of 186 target gene sets associated with at least one predictor gene set; 20 and 24 out of these 154 gene sets had associations only in 25T and in 25N, respectively. In the BioCarta database, there were 193 of 217 target gene sets associated with at least one predictor gene set; 16 and 21 out of these 193 gene sets had associations only in 25T and 25N, respectively.

**Figure 6:**
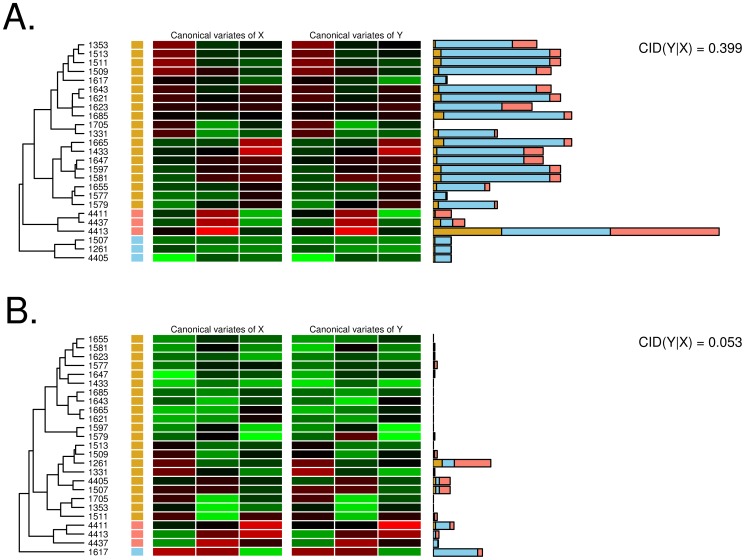
Example of GSAA using the CID. Heatmaps for the first three canonical variates of genes in the predictor gene set (X) and those of genes in the target gene set (Y) (i.e., the graft versus host disease pathway) for each subgroup were shown in the left and center panels. The weighted squared discrepancies between the marginal and conditional cdfs evaluated for one sample were indicated by the widths of the bars in the right panel. (A) Biosynthesis of amyotrophic lateral sclerosis als (related) pathway. (B) Selenoamino acid metabolism (unrelated) pathway.

Figure 6 provided examples for our attempt at relating the statistical significance of the CID to possible biological meanings. In these examples, the set of 13 genes in the graft versus host disease pathway in the KEGG database was used as the target (*Y*). We compared the CID results when the twenty-eight genes in the amyotrophic lateral sclerosis als pathway (Figure 6A) and the forteen genes in the selenoamino acid metabolism pathway (Figure 6B) were set to be the predictor (*X*), respectively. The former pathway (hereafter referred to as ‘related pathway’) yielded the largest value of the adjusted CID (i.e., 4.350, corresponding to a CID value 0.399) and the latter pathway (hereafter referred to as ‘unrelated pathway') had a relatively small adjusted CID (i.e., -1.539, corresponding to a CID value 0.053). The expression of genes in related pathways showed the splits of the cohort into three subgroups of 19, 3, and 3 arrays, respectively, by hierarchical clustering (labeled as brown, blue, and pink in Figure 6A), whereas expression of genes in unrelated pathways divided 25T into three subgroups of 21, 3, and 1 arrays (labeled as brown, pink, and blue in Figure 6B). To reduce the computational complexity, we consider up to the first three canonical variates from both gene sets for the CID estimation.

Heatmaps for the first three canonical variates of the target gene set and those of the predictor gene set in each subgroup were shown in Figure 6. The marginal and conditional distributions of the target gene sets were evaluated accordingly. The weighted squared discrepancies between the marginal and conditional cdfs (i.e., Equation (4) in ‘Materials and Methods’ Section) evaluated for one sample were indicated by the widths of the bars in the right panel of Figure 6. The discrepancy was noticeably large if the predictor gene set was claimed to be associated with the target gene set (Figure 6A, right panel). However, for the unrelated pathway, most of the conditional cdfs were similar to the marginal cdf and resulted in a small discrepancy and, therefore, a small CID value (Figure 6B, right panel). In the related pathway, the subgroup labeled in blue contributed 75.82% to the CID value (Figure 6A, right panel). By observing the heatmap of three arrays in this subgroup (Figure 6A, left panel), one can see that the expression levels in this subgroup were relatively homogeneous with regard to genes; that is, the three canonical variates in Array ID 1507, 1261, and 4405 were all relatively low. When evaluating such homogeneous subgroups using Equation (3), larger values of the conditional cdf were usually produced. This kind of homogeneity was not obvious in unrelated pathways.

### Supervised gene set association analysis using the CID

Here, the CID was adopted for identifying the functional expression of a whole set of signaling molecules (gene set 2) to be significantly associated with the given transcription factors (gene set 1). The analytical flowchart has been outlined in Figure 2 (see the ‘Materials and Methods’ section).

**Figure 7:**
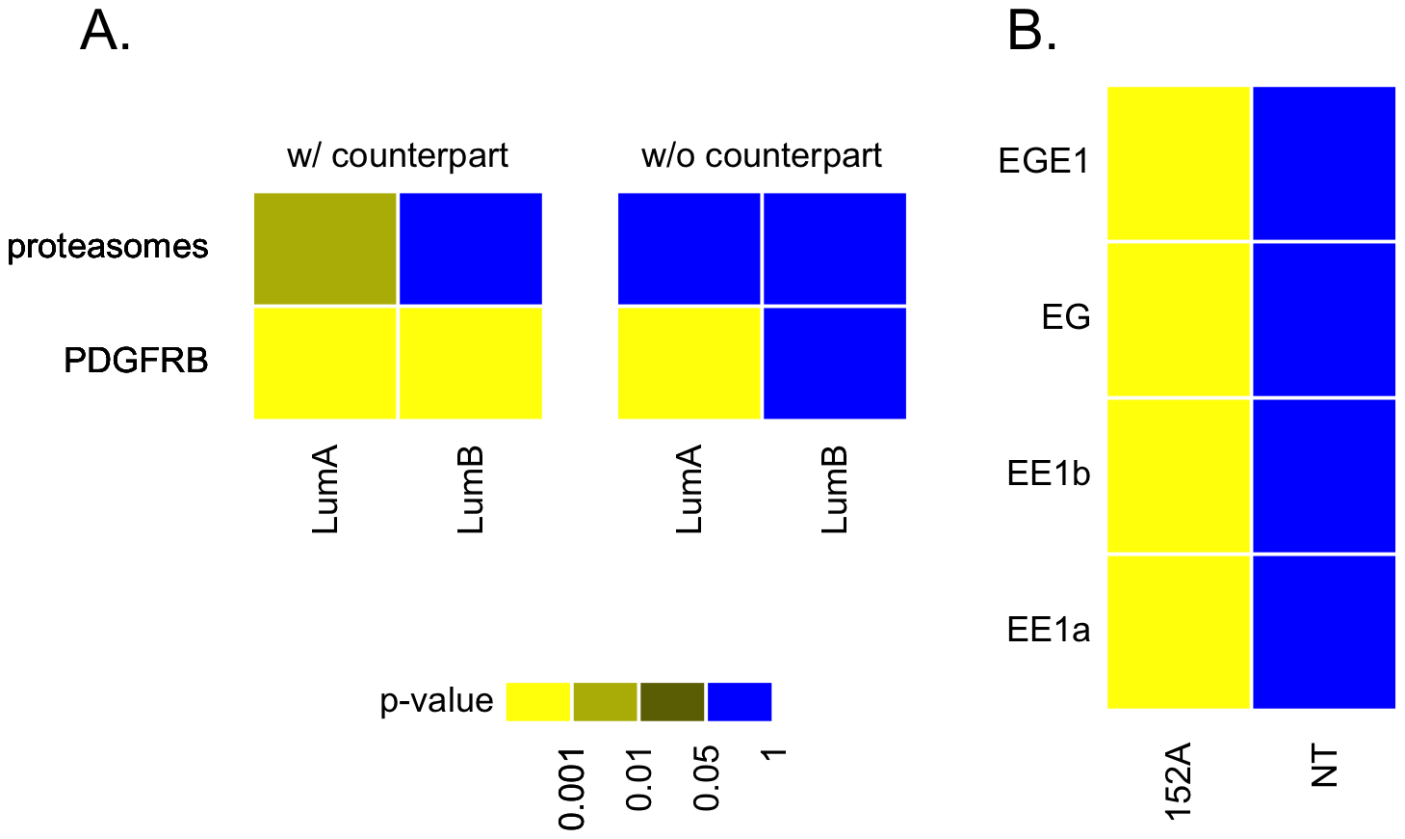
Supervised GSAA on two selected gene sets that show potential gene co-expression relationship in a given population. Panel A shows the first example for the co-existing gene expression relationship between transcription factors and a signal transduction pathway of interest. *MYC* and *STAT3* are the two chosen transcription factors. Both proteasomes and PDGFRB signal transduction pathways were selected for predicting their gene co-expression relationship with *MYC* and *STAT3* via GSAA in a cohort, respectively. Lumial A and luminal B are the two cohorts. Panel B shows the second example for the co-existing gene expression relationship between transcription factors and a subnetwork of interest. ESR1(E), GATA3(G) and E2F1(E1) are the three transcription factors of choice based on their combinatorial co-expression relationships with the published gene sets [19]. The designation for each co-expression relationship is indicated next to the results. For instance, the final labeled names for the gene sets of subnetworks a, b and transcription factors ESR1 and E2F1 (EE1) have been designated as EE1a, EE1b. 152A stands for the dataset from a cohort including 61 group IE and 91 ER(-) breast cancer patients. NT stands for another sample including 25 non-tumor parts of the breast cancer patient sample.

Two examples were illustrated in this study. The first example, performing the GSAA analysis using the CID (Figure 7A), demonstrated that a significant dynamical change of a signaling transduction pathway (STP) could possibly be due to the co-existence of two transcription factors (TFs). The predictor consists of two transcription factors, *MYC* and *STAT3*, while the gene sets in the proteasomes_STP and PDGFRB_STP were the targets. When a specific population with low expression levels of *MYC* and *STAT3* appears to be the counterpart of cancer subtypes, such as, ERBB2+, we observed that PDGFRB_STP co-expressed with aberrantly expressed *MYC* and *STAT3* in two tumor subtypes, luminal A and luminal B (*p*-values ≤ 0.001 in Figure 7A left panel). When the tumor subtypes without a counterpart were analyzed via GSAA, we observed PDGFRB_STP co-expressed with aberrantly expressed *MYC* and *STAT3* only in luminal A (Figure 7A right panel). The GSAA between the proteasomes_STP and two transcription factors, *MYC* and *STAT3*, was performed simultaneously by using the cohort with a counterpart or without a counterpart for two cohorts, respectively. Luminal A has relatively higher mRNA levels of both *MYC* and *STAT3* than luminal B does. Therefore, the co-expression event between these two gene sets is hypothesized to be primarily in luminal A. GSAA results suggest that the most relevant proteasomes_STP will be co-expressed with *MYC* and *STAT3* in luminal A not in luminal B (Figure 7A).

In the second example, we picked four subnetworks to be analyzed by GSAA to show the co-expression of the network components with selected TFs. This example demonstrated GSAA to be powerful in hunting for the potential regulators of a given gene signature. *ESR1* (E), *GATA3* (G) and *E2F1* (E1) were the three transcription factors of choice based on their combinatorial co-expression relationships with the published gene sets (Table 2) [19]. The TFs were found relevant to the previously predicted network components in all subnetworks using the subcohort of 61 group IE (ER(+) and PR(+)) and 91 ER(-) breast cancer patients (152A) but not using the subcohort of 18 and 7 non-tumor samples (NT) from ER(+) and ER(-) patients, respectively (Figure 7B). The *p*-values were all less than 0.001 in 152A whereas the *p*-values in NT were all greater than 0.05. Both 152A and NT, which combined part of the ER(+) and ER(-) subcohorts, were heterogeneous in nature.

## Discussion

The main goal of this study is to identify differential association between the pair of gene sets based on a predefined collection of gene sets using the gene expression data. In our previous work, gene expression relationship between a transcription factor and a target gene has been established by combining both univariate CID and the correlation coefficient [17]. We further developed a bivariate CID as a simple version of the multivariate space of the transcriptional regulatory network [19]. In this study, the CID serves as a statistical measure to quantify partial linear and non-linear relationship between two gene sets. From the numerical results of the synthesized data set, we found that the proposed method provides a robust and powerful statistical framework for identifying linear or non-linear association between gene sets.

The distribution-based methods, CID, KLD and HD, adopt a similar concept of dependence by measuring discrepancy between the marginal and conditional distributions. However, KLD and HD were much less powerful than the CID. This might be due to the fact that more information loss had occurred during the estimation of the probability density functions (pdfs) for KLD and HD than during the estimation of the cumulative distribution functions (cdfs) for the CID. For each observation in the sample, the estimation of cdfs was independent, whereas the estimation of pdfs relying on subgrouping produced only one estimate for all observations in the same subgroup. The former introduced variability into the estimation from which the CID can more precisely differentiate different levels of association. Therefore, results showed that the CID has a higher power than KLD and HD. The estimation of cdfs is technically easier to compute than the estimation of pdfs, whose precision might also be altered by using different methods of subgrouping.

When applied to a breast cancer microarray dataset, the results reveal that our approach could discover pairs of gene sets with enriched associations hidden in the data. In addition, the identified gene set associations may be useful in the regulation or network construction of gene sets, and they can also be used to investigate different co-expression patterns found in different clinical cohorts. Here, the GSAA using a multivariate CID suggested a bottom-up approach for identifying the functional expression of a whole set of signaling molecules (gene set 2) to be significantly associated with the given transcription factors (gene set 1) (Figure 7). In the first example of supervised GSAA, we had demonstrated that the PDGFRB signal transduction pathway (PDGFRB_STP) was differentially regulated by *STAT3* and *MYC* in non-tumor and tumor components (Figure 7A), which was supported by our previous studies ([33] and unpublished data of ours). In the second example, the results suggested that expression profiles of the target gene sets follow a consensus pattern of dynamical changes in NT (Figure 7B). However, the consensus feature was not found in 152A.

This GSAA could be less hypothesis driven and less steps required in uncovering the potential interactions between two gene sets of interest. The proposed CID aims to discover the target gene sets whose expression patterns follow a consensus pattern of dynamical changes in a population. Therefore, GSAA may not be sensitive to those populations with little dynamical changes in the gene expression patterns of two gene sets. In addition, the heterogeneous nature of cancer is more likely to make difficulties in finding the consensus feature in a cohort to be reproducible in another cohort. Therefore, it is recommended to combine a tumor cohort with its counterpart to enrich the expression patterns so that the false detection rate can be significantly reduced by eliminating the confounder effect.

In conclusion, we have developed a methodology for extracting multivariate associations by using the coefficient of intrinsic dependence (CID). It is more powerful especially when the type of association was present in a form of non-linearity or variation. To date, most of the methods developed for GSAA have focused on the statistical tests of association of phenotypes rather than on the inter-gene set correlations. Our approach has the potential to construct a statistically relevant network from microarray data, and it can be used to complement the conventional gene set analysis which is only interested in identifying gene sets associated with the studied phenotypes.

## Supporting Information

Figure S1 **True positive rate under different level of association for CID using kmeans and SOM for subgrouping in the multivariate normal model for p = 5 and q = 2.**
(PDF)

Table S1 **GEO accession numbers of tumor and nontumor samples used in this study.**
(PDF)

Table S2 **The mapping results of Entrez ID from GSEA website to Agilent feature numbers using DAVID (Huang et al., 2008, 2009).**
(PDF)

Table S3 **GEO accession numbers of the samples used in the supervised GSAA.**
(PDF)

Table S4 **The genes in the signaling transduction pathways (STPs) of interest in the analysis of the supervised GSAA.**
(PDF)

Table S5 **Significant associated pathways in KEGG database using 25 tumor samples (25T) and 25 nontumor samples (25N).** The rows are predictors and the columns are the targets. '11' (yellow) denotes 18 significant associations in both 25T and 25N. '10' (red) denotes 380 significant associations in 25A but not in 25N. '1' (green) denotes 2724 significant associations in 25N but not in 25T.
(PDF)

Table S6 Significant associated pathways in BioCarta database using 25 tumor samples (25T) and 25 nontumor samples (25N). The rows are predictors and the columns are the targets. '11' (yellow) denotes 34 significant associations in both 25T and 25N. '10' (red) denotes 692 significant associations in 25A but not in 25N. '1' (green) denotes 693 significant associations in 25N but not in 25T.
(PDF)
