# Supplementary material for: Identifying Gene Set Association Enrichment Using the Coefficient of Intrinsic Dependence
Source: PLoS One. 2013 Mar 14;8(3):e58851. doi: 10.1371/journal.pone.0058851 (PMC3597597; doi:10.1371/journal.pone.0058851)

Figure S1. True positive rate under different level of association for CID using kmeans and SOM for subgrouping in the multivariate normal model for p = 5 and q = 2.

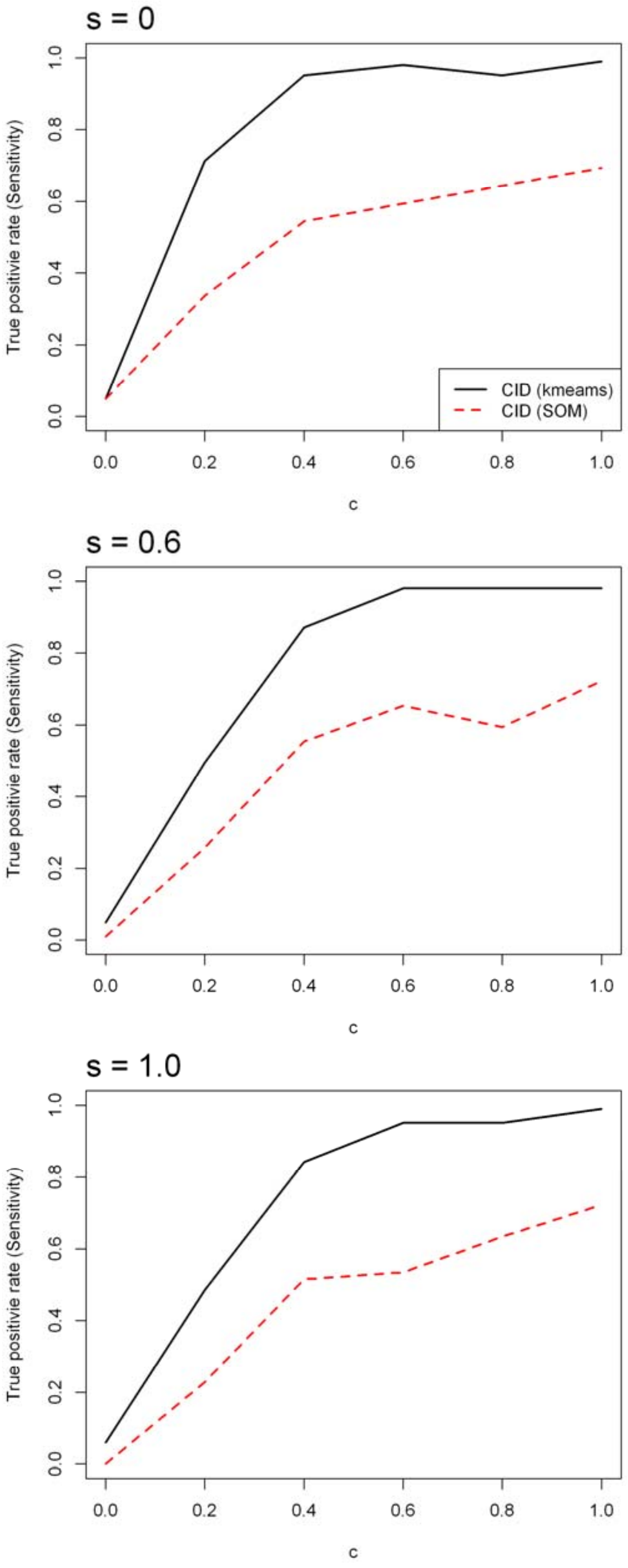

Supplement: Figure S1 — True positive rate under different level of association for CID using kmeans and SOM for subgrouping in the multivariate normal model for p = 5 and q = 2. (PDF) [file pone.0058851.s001.pdf]
