# Supplementary material for: Identifying Gene Set Association Enrichment Using the Coefficient of Intrinsic Dependence
Source: PLoS One. 2013 Mar 14;8(3):e58851. doi: 10.1371/journal.pone.0058851 (PMC3597597; doi:10.1371/journal.pone.0058851)
Supplement: Table S1 — GEO accession numbers of tumor and nontumor samples used in this study. (PDF) [file pone.0058851.s002.pdf]

Table S1. GEO accession numbers of tumor and nontumor samples used in this study.

Accession	Description
GSM237162	breast nontumor part_N_rep1_1539
GSM237163	breast nontumor part_N_rep1_1541
GSM237164	breast nontumor part_N_rep1_1543
GSM237171	breast nontumor part_N_rep1_1591
GSM237172	breast nontumor part_N_rep1_1593
GSM593690	breast nontumor part_N_rep1 5293
GSM593691	breast nontumor part_N_rep1 5403
GSM593692	breast nontumor part_N_rep1 5405
GSM593693	breast nontumor part_N_rep1 5407
GSM593694	breast nontumor part_N_rep1 5409
GSM593695	breast nontumor part_N_rep1 5411
GSM593696	breast nontumor part_N_rep1 5413
GSM593697	breast nontumor part_N_rep1 5415
GSM593698	breast nontumor part_N_rep1 5417
GSM593699	breast nontumor part_N_rep1 5419
GSM593700	breast nontumor part_N_rep1 5421
GSM593701	breast nontumor part_N_rep1 5423
GSM593702	breast nontumor part_N_rep1 5425
GSM593703	breast nontumor part_N_rep1 5427
GSM593704	breast nontumor part_N_rep1 5429
GSM593705	breast nontumor part_N_rep1 5431
GSM593706	breast nontumor part_N_rep1 5433
GSM593707	breast nontumor part_N_rep1 5435
GSM593708	breast nontumor part_N_rep1 5437
GSM593709	breast nontumor part_N_rep1 5999
GSM237155	breast tumor part_IDC_rep1_1509
GSM237156	breast tumor part_IDC_rep1_1511
GSM237157	breast tumor part_IDC_rep1_1513
GSM237166	breast tumor part_IDC_rep1_1579
GSM237167	breast tumor part_IDC_rep1_1581
GSM237165	breast tumor part_IDC_rep1_1577
GSM237139	breast tumor part_IDC_rep1_1261
GSM237141	breast tumor part_IDC_rep1_1331
GSM237143	breast tumor part_IDC_rep1_1353
GSM237148	breast tumor part_IDC_rep1_1433
GSM237154	breast tumor part_IDC_rep1_1507
GSM237174	breast tumor part_IDC_rep1_1597
GSM237176	breast tumor part_IDC_rep1_1617
GSM237178	breast tumor part_IDC_rep1_1621
GSM237179	breast tumor part_IDC_rep1_1623
GSM237180	breast tumor part_IDC_rep1_1643
GSM237182	breast tumor part_IDC_rep1_1647
GSM237186	breast tumor part_IDC_rep1_1655
GSM237190	breast tumor part_IDC_rep1_1665
GSM237194	breast tumor part_IDC_rep1_1685
GSM593751	breast tumor part_IDC_rep1 4437
GSM593737	breast tumor part_IDC_rep1 4405
GSM593741	breast tumor part_IDC_rep1 4413
GSM593740	breast tumor part_IDC_rep1 4411
GSM237199	breast tumor part_IDC_rep1_1705
