# Supplementary material for: Identifying Gene Set Association Enrichment Using the Coefficient of Intrinsic Dependence
Source: PLoS One. 2013 Mar 14;8(3):e58851. doi: 10.1371/journal.pone.0058851 (PMC3597597; doi:10.1371/journal.pone.0058851)
Supplement: Table S2 — The mapping results of Entrez ID from GSEA website to Agilent feature numbers using DAVID (Huang et al., 2008, 2009). (PDF) [file pone.0058851.s003.pdf]

Table S2. The mapping results of Entrez ID from GSEA website to Agilent feature numbers using DAVID (Huang et al., 2008, 2009).

Pathway	AgilentFeatureNumber
KEGG_GLYCOLYSIS_GLUCONEOGENESIS	19460;4536;5955;13234;5508;16372;434;7126;14852;12004;102;15872;7214;9521;6135;10159;14715;17382;6404;20259;9736;4768;16961;7529;8024;21797;4042;16996
KEGG_CITRATE_CYCLE_TCA_CYCLE	5955;13234;3982;16309;7960;9578;1117;8389;7529;19365;15798;2509;3012;3982;21524
KEGG_PENTOSE_PHOSPHATE_PATHWA	3643;5508;6135;10159;19113;16372;20317;14647;2095;8024;10929;16996;12771;102
KEGG_PENTOSE_AND_GLUCURONATE_INTERCONVERSIONS	17849;19230;17515;10674;14369;13229;11227;22286;21110;21190;16804
KEGG_FRUCTOSE_AND_MANNOSE_METABOLISM	2566;2782;6135;20588;10159;5678;1176;2657;17037;8301;16372;434;8024;13052;21110;102;9541
KEGG_GALACTOSE_METABOLISM	19460;3279;5508;1716;10159;13608;434;21797;2242;2715;4042;11227;16996;21110;2071;102
KEGG_ASCORBATE_AND_ALDARATE_METABOLISM	7214;9521;2253;19230;4768;13229;22286;21190;16804
KEGG_FATTY_ACID_METABOLISM	6508;1920;7214;9521;19877;387;21835;19499;6404;7126;5647;4768;4337;6292;13602;10950;20499;2855;1157
KEGG_STEROID_BIOSYNTHESIS	21506;15770;13524;4744;1086;8536;13760;21601;15604
KEGG_PRIMARY_BILE_ACID_BIOSYNTHESIS	17365;13272;9614;156;20885;21147;19480;18538;5337;3084
KEGG_STEROID_HORMONE_BIOSYNTHESIS	20761;2523;6418;18538;17820;10537;2870;4217;17830;11058;14697;8638;13229;19973;331;9556;5078;19230;5630;21307;9614;22286;21190;16804
KEGG_OXIDATIVE_PHOSPHORYLATION	3654;15677;17829;12462;13919;15741;5554;16221;1629;15741;15741;21216;11918;16472;17494;18278;1274;1416;4031;11658;20873;9225;16135;17070;10966;15482;6654;6583;9900;17653;18233;4303;7960;9578;1834;11354;6658;13361;13256;11023;14731;3249;12539;11590;8462;9434;20586;20911;3329;20703;22293;15297;17520;12482;304;16579;239;8742;4087;5365;2111;12216;15466;6498;13016;3740;5250;1159;17435;6837;7096;22368;18951;
KEGG_PURINE_METABOLISM	6774;12689;20336;11459;20146;12705;8478;17172;15290;6610;1670;6441;13197;1670;1670;21382;16377;4017;1809;22382;18061;18962;8153;7106;6364;C12707.5;134;17410;9293;12905;12932;13565;21331;19837;13764;4661;19006;20757;11488;17605;22033;4325;10983;10827;18483;14439;13709;6125;13290;1944;8097;20897;20622;4192;3415;11131;12614;15800;13282;1191;14852;5293;2039;17903;16361;1014;14574;8617;16353;18751;4579;3913;8968;12540;9802;4105;15614;4499;12732
KEGG_PYRIMIDINE_METABOLISM	12689;6774;11459;20146;12705;17172;8041;11614;15290;6610;1808;1670;1670;1670;4017;16377;1809;7106;C12707.5;9293;17622;8539;19006;22033;10983;10827;13709;14439;6125;7477;15519;9589;20897;20622;10247;9429;11645;15800;5293;16361;14574;8617;16353;18751;11648;7871;21427;4579;21680;15585;12540;15614;4499
KEGG_ALANINE_ASPARTATE_AND_GLUTAMATE_METABOLISM	14033;1356;18353;C10362.2;12924;5558;11123;22205;21182;3077;13764;5418;653;14994;16278;683;4105;2958
KEGG_GLYCINE_SERINE_AND_THREONINE_METABOLISM	3479;18268;1356;15054;13603;16052;5861;5577;17450;21732;21742;3683;3250;15696;7529;16278;22125;17608;8901;18807;8640
KEGG_CYSTEINE_AND_METHIONINE_METABOLISM	21972;6361;16052;7786;14715;22205;20259;16248;14994;15359;683;8155;8168;8901;8577;18807;22071;4056
KEGG_VALINE_LEUCINE_AND_ISOLEUCINE_DEGRADATION	14714;7214;9521;19877;22205;21835;20139;19499;16485;8587;12311;5647;4768;3211;13935;7529;21556;16259;10242;2018;261;17211;3404;19398;5811
KEGG_VALINE_LEUCINE_AND_ISOLEUCINE_BIOSYNTHESIS	5955;6674;16644;13234;379;17211;3404;12818
KEGG_LYSINE_DEGRADATION	8532;21810;7214;9521;15495;19877;10884;21835;16676;9432;5647;4768;12710;3498;15798;4337;21241;9117;10667;12263;20531;8640
KEGG_ARGININE_AND_PROLINE_METABOLISM	6361;14033;22431;21825;12924;5558;11123;3287;10228;319;3711;20273;14994;12950;14197;683;7446;15379;21972;18268;C10362.2;20850;7214;13603;9521;16854;4347;131;9003;4768;3287;11737;13626;7334;3257
KEGG_HISTIDINE_METABOLISM	1686;18268;7214;9203;9521;3157;1118;9736;5418;4768;3039;12784;22326;2958;13868;3749
KEGG_TYROSINE_METABOLISM	18268;4672;14714;15054;13061;9203;2523;5861;3157;22205;12675;1118;6404;13007;9736;7126;14994;3039;12784;683;1275;16001;7899;21838;13868
KEGG_PHENYLALANINE_METABOLISM	9736;18268;4672;14994;15054;5861;683;1275;5208;7899;22205;1118
KEGG_TRYPTOPHAN_METABOLISM	18268;14714;7214;9521;14239;17131;4065;19877;6298;22205;21835;4299;1118;9556;5078;5647;4768;2041;15798;4337;4484;7074;402;10667;7140;19
KEGG_BETA_ALANINE_METABOLISM	1686;6361;5647;4768;653;15054;7214;9521;15519;5861
KEGG_TAURINE_AND_HYPOTAURINE_METABOLISM	12324;13272;653;15359
KEGG_SELENOAMINO_ACID_METABOL	9203;3157;2188;3017;12614;3039;12784;3618;12324;8168;13868;8901;22071;18807
KEGG_GLUTATHIONE_METABOLISM	6361;9608;6226;12675;14647;5449;21066;4555;12950;2202;18282;18680;11456;12910;18831;11069;16637;5108;16353;18207;11156;4213;1994;22225;2213;12513;4189;12324;3257
KEGG_STARCH_AND_SUCROSE_METABOLISM	20336;19460;5508;18424;13608;9901;434;5036;17515;13933;13229;1961;21646;22427;19230;14493;21797;4042;11227;16996;22286;21190;2071;16804
KEGG_N_GLYCAN_BIOSYNTHESIS	11230;22275;19963;17281;15939;8144;21747;10527;9238;18153;4565;1372;10815;14180;13422;21017;17176;7482;20742;19985;3744;4870;5641
KEGG_OTHER_GLYCAN_DEGRADATIO	4620;10709;20227;15744;13542;4929;15103;578
KEGG_O_GLYCAN_BIOSYNTHESIS	3337;17055;5197;5136;8905;17467;4157;18391;1074;6006;18941;10923;17369;9497;19490;457;17157;18413;15940;17467;1143;350;21597
KEGG_AMINO_SUGAR_AND_NUCLEOTIDE_SUGAR_METABOLISM	19460;3279;18353;2566;2782;5508;9991;20588;18424;18175;5678;12020;578;434;17886;4886;5521;7064;12611;2715;11227;14502;13052;16996;9541
KEGG_GLYCOSAMINOGLYCAN_DEGRADATION	7891;18408;17515;4110;6030;18731;13804;17360;14649;14651;6656;3391;578;21028
KEGG_GLYCOSAMINOGLYCAN_BIOSYNTHESIS_CHONDROITIN_SULFATE	13700;13103;10296;6988;14013;19180;6099;15671;1870;12063;6029;21697;6559;3400
KEGG_GLYCOSAMINOGLYCAN_BIOSYNTHESIS_KERATAN_SULFATE	15228;1143;350;5302;6486;5641
KEGG_GLYCOSAMINOGLYCAN_BIOSYNTHESIS_HEPARAN_SULFATE	20235;206;1796;6988;14013;17029;5300;13103;8984;12609;6381;16237;1870;6559;18324;12823
KEGG_GLYCEROLIPID_METABOLISM	6464;13861;16542;14246;2242;13118;21110;18511;5530;3888;7211;13120;3479;7214;9521;19125;8228;17495;18979;4694;4768;2542;6981;3766;12842;13760;5673;6821
KEGG_INOSITOL_PHOSPHATE_METABOLISM	9746;12542;22517;445;2253;3054;21353;3148;15037;15653;21701;505;12421;14660;14103;2681;17762;16485;581;10935;2341;3804;16847;8721;15218;15538;7530;4399;5941;20355
KEGG_GLYCOSYLPHOSPHATIDYLINOSITOL_GPI_ANCHOR_BIOSYNTHESIS	11328;9092;18889;9238;4239;20277;12285;12886;6578;19273;20959
KEGG_GLYCEROPHOSPHOLIPID_METABOLISM	384;11426;15592;8664;4103;8356;10339;13861;16542;14246;14557;21022;4336;7570;10926;C10773.4;14717;15377;21022;13118;18061;21112;19125;17495;8228;20950;18979;4682;15391;2254;4694;6981;3766;11854;15799;12842;19686;14633;9682;5673;8923;1603
KEGG_ETHER_LIPID_METABOLISM	17495;4103;10339;14557;21022;7031;7570;2090;4336;13204;10926;C10773.4;14717;15377;21022;20112;6981;3766;11854;15799;7263;14633;19686;96
KEGG_ARACHIDONIC_ACID_METABOLISM	6220;1785;14132;945;10339;17219;11329;15929;21022;4336;7570;10926;C10773.4;14717;15377;21022;4757;11456;12960;9016;11156;1994;22225;12324;16993;16993;15799;14633
KEGG_LINOLEIC_ACID_METABOLISM	1785;945;10339;17037;11329;21022;4336;7570;10926;C10773.4;14717;15377;21022;15799;4757;14633;12960;19973
KEGG_ALPHA_LINOLENIC_ACID_METABOLISM	21022;7570;4336;10926;C10773.4;14717;15377;21022;6292;10339;15799;14633
KEGG_SPHINGOLIPID_METABOLISM	22412;1145;4388;15744;17495;17754;8808;7544;1272;22210;4992;6845;14473;2242;3463;20051;6981;3766;1405;472;11915;2304;20051;17803
KEGG_GLYCOSPHINGOLIPID_BIOSYNTHESIS_LACTO_AND_NEOLACTO_SERIE	1149;C8894;11159;12844;14071;19057;21669;14869;2596;18488;6486;18224
KEGG_GLYCOSPHINGOLIPID_BIOSYNTHESIS_GLOBO_SERIES	2242;12844;1143;3;350;2596;578
KEGG_GLYCOSPHINGOLIPID_BIOSYNTHESIS_GANGLIO_SERIES	20791;13956;18780;15928;14063;1143;350;578;18337
KEGG_PYRUVATE_METABOLISM	15274;5955;7214;13234;9521;19877;14715;8465;20259;4240;19677;4768;10895;7529;19365;2509;19324;6365;21110;14852;16279
KEGG_GLYOXYLATE_AND_DICARBOXYLATE_METABOLISM	3479;22482;19365;2827;12266;21907;21524
KEGG_PROPANOATE_METABOLISM	7214;9521;3982;19877;14715;20139;16485;20259;5647;4768;3211;21556;3012;10242;6365;3982;19028
KEGG_BUTANOATE_METABOLISM	5955;16457;13234;7214;9521;19877;21835;17971;13874;8587;17037;5647;4768;14322;653;13935;1957;16251
KEGG_ONE_CARBON_POOL_BY_FOLAT	11131;8041;22326;2063;17813;17737;5577;8155;12266;21382;22339;21742
KEGG_RIBOFLAVIN_METABOLISM	15080;20336;2657;6008;4591
KEGG_NICOTINATE_AND_NICOTINAMIDE_METABOLISM	6774;20336;2501;14714;9905;15800;16787;18751;1369;20897;15614;7139;22208;10971

KEGG_PANTOTHENATE_AND_COA_BIOSYNTHESIS	20650;20336;20684;12358;17943;12575;15519;15705;17211;3404
KEGG_FOLATE_BIOSYNTHESIS	6556;20299;17737;5656;16021;9371
KEGG_RETINOL_METABOLISM	14065;4009;18926;11329;7126;12387;13703;13229;4757;12960;7600;19973;18841;7211;1091;4449;9556;6404;19230;14093;22286;21190;16804
KEGG_PORPHYRIN_AND_CHLOROPHYLL_METABOLISM	17653;1174;20258;1220;1256;7397;19230;17515;2037;17664;13229;9416;22286;21190;7686;5767;8750;8031;8820;16804
KEGG_TERPENOID_BACKBONE_BIOSYNTHESIS	20054;5866;13935;17938;19877;7930;11279;22391;1167
KEGG_LIMONENE_AND_PINENE_DEGRADATION	7899;5647;4768;7214;9521
KEGG_NITROGEN_METABOLISM	13398;15121;496;14033;19981;C10362.2;10594;16517;12924;2909;8649;116;5558;18807
KEGG_SULFUR_METABOLISM	17820;12614;6099;6418;6029;3400
KEGG_AMINOACYL_TRNA_BIOSYNTHE	20209;7709;3673;16644;9861;4065;2826;18199;19987;170;19120;6674;4083;2041;12459;379;3915;12818;3618;17813;8719;7560;15310;2398;8031;565
KEGG_METABOLISM_OF_XENOBIOTICS_BY_CYTOCHROME_P450	1785;21805;9608;6226;12675;11329;7305;7126;5449;21066;4555;2202;18282;18680;13229;4757;12910;12960;11069;19973;16637;9556;6404;5078;19230;9736;14369;4189;22286;21190;16804
KEGG_DRUG_METABOLISM_CYTOCHROME_P450	1785;9608;6226;12675;11329;7126;5449;21066;4555;10894;2202;2759;19218;18282;18680;13229;4757;12910;12960;11069;7600;19973;16637;18268;14714;6404;19230;9736;4189;22286;21190;16804
KEGG_DRUG_METABOLISM_OTHER_ENZYMES	8478;15519;1944;10247;3415;11645;17515;13229;11132;7600;19973;16361;11648;7871;21427;21680;15585;19230;10551;15402;22286;21190;7477;16804
KEGG_BIOSYNTHESIS_OF_UNSATURATED_FATTY_ACIDS	2023;13272;15811;6292;12376;9751
KEGG_ABC_TRANSPORTERS	19667;7924;14390;19754;6840;9681;3295;13916;13466;20036;10685;12853;7755;19421;5950;21038;6147;18827;2692;3973;12661;3446;13454
KEGG_RIBOSOME	8345;7440;6148;9383;20735;10243;6369;17715;12795;1976;12922;6794;19619;9855;3452;5014;14145;13126;13474;4113;15767;11733;3465
KEGG_RNA_DEGRADATION	16779;10864;18863;6610;13535;C10627.6;14733;15813;16914;18799;15642;12004;12345;5048;4841;2072;891;7029;14570;15475;17382;8260;6758;19921;10909;22493
KEGG_RNA_POLYMERASE	12689;11459;20146;12705;14574;17172;20622;4579;4017;10827;1809;14439;6125
KEGG_BASAL_TRANSCRIPTION_FACTO	7367;11683;13049;11724;17370;2682;5915;6616;14459;6714;18666;14909;12556;11618;19423;13968;15462;3395;1019;17000
KEGG_DNA_REPLICATION	8617;C12707.5;9293;9943;1474;3210;8986;21683;3500;13872;19006;7602;2749;6838;22033;2221;10983;17514;14990;3332
KEGG_SPLICEOSOME	16779;17388;14940;1479;14733;21230;458;5981;908;896;15456;21484;11956;875;22503;2072;9886;891;6293;3783;15837;6290;681;15776;3203;12322;2174;3409;19957;16089;13408;2529;6058;19428;13814;20274;3778;19606;11556;2604;2394;16558;5891;16408;15475;798;14467;3609;782;10563;13561;13086;12331;4400;11051;4084;2888;1864;16354
KEGG_PROTEASOME	14704;8213;6493;16498;20474;2880;13893;15618;3555;17283;14330;8132;5162;20090;1293;15647;10775;1815;12348;7124;2792;10706;8278
KEGG_PROTEIN_EXPORT	18922;21820;495;18774;13790;16196;19412;9910;2526;1800;1910;17444
KEGG_PPAR_SIGNALING_PATHWAY	17365;21853;18538;16014;12376;10820;12966;2625;4240;17197;3935;10545;5357;2166;21147;6292;19480;10950;13036;13120;21968;6508;1920;20885;1974;15690;4028;13935;17643;14508;17238;16366;2484;13602;1157
KEGG_BASE_EXCISION_REPAIR	8617;9293;10532;13872;19342;19006;9051;15381;6003;22033;10983;15245;20179;3332;14755
KEGG_NUCLEOTIDE_EXCISION_REPAIR	6040;8617;15751;934;6467;9293;17063;9943;1474;3210;3869;11142;20907;8986;3500;13872;16808;19006;18716;22033;10983;14990;2889;12556;7820;3332;11780;1019
KEGG_MISMATCH_REPAIR	22033;15551;9636;14990;4702;9293;9943;3332;1474;3210;19492;8986;13872;3500;C12687.1
KEGG_HOMOLOGOUS_RECOMBINATIO	6312;19879;329;17190;12612;3286;9293;2813;3210;10860;8986;3500;10343;14908;22033;7986;7124;C10009;20363
KEGG_NON_HOMOLOGOUS_END_JOINI	17946;6003;8210
KEGG_MAPK_SIGNALING_PATHWAY	14573;16911;8000;5373;9404;2555;18485;21944;10344;13696;12157;4458;19099;11089;13817;15977;8554;18828;14252;21589;4596;1378;9572;10712;7638;9421;17955;14482;14633;5142;414;2747;1209;11339;18383;19188;22250;22509;15172;10090;1748;12636;9739;10926;534;C10773.4;10577;14717;1873;16547;11290;8849;2109;13522;3699;10662;22278;6410;20580;19712;14930;12613;8898;6700;18427;7033;9028;10119;13486;11881;7252;9174;13089;11466;16304;20912;1207;21354;1668;7046;19366;6865;21022;7570;4336;3877;9217;21022;20533;6756;18417;1842;16364;2490;20323;4048;20379;6034;1731;21087;18959;9099;17977;15799;6879;10456;13860;18255;5386;21235;17428;7993;13078;10339;15669;2529;6058;22401;22172;15377;21637;15176;5668;19606;13634;17571;10553;11352;13384;542;3523;20986;12299;20135;9110;13150;13647;7223
KEGG_ERBB_SIGNALING_PATHWAY	14573;10006;21235;5720;7993;11559;22399;5870;5848;15669;18091;10090;1748;9217;1873;11290;4458;21517;6700;21562;2703;17571;8055;14863;15779;10119;11352;14354;542;4473;1924;3851;194;2446;21087;8592;15218;15538;7530;17977;4399;5941;446
KEGG_CALCIUM_SIGNALING_PATHWAY	3894;2501;6301;18837;4634;18952;18635;1668;8516;11855;20728;1652;7544;885;6865;2512;21353;11089;13510;18862;12926;8510;18828;21979;17762;15802;12932;2893;5919;19837;14785;11488;4544;194;2446;6153;18959;8327;11929;5316;2726;4399;5941;20355;11982;22008;18587;12542;22517;7993;445;5862;16653;15159;13876;4192;15669;17280;319;22401;19232;14551;3711;20273;17004;21637;15176;15590;14660;14103;20582;2681;3343;19994;16426;4669;3428;4388;10583;5799;4331;16800;8437;1279;17540;10466;15639;11061;9905;2901;11466;16304;3614;15355;14734;7223
KEGG_CYTOKINE_CYTOKINE_RECEPTOR_INTERACTION	19740;9552;5189;5622;2342;7864;3553;8740;21803;1367;21035;21587;12665;12665;14748;4035;1579;17187;19321;3485;14986;17483;1524;9639;7380;13571;11835;4226;18669;14774;17453;19478;11130;10908;1378;9572;12921;17955;19852;1169;5285;15943;10028;20853;12856;11925;10428;281;9042;278;1469;15172;7618;2462;17987;9400;8351;5370;16755;10422;6790;22445;14829;3349;188;12785;11671;16291;2440;10451;13843;14134;6865;5958;14588;20533;20469;7618;4149;12637;16422;9232;2552;6034;9883;21326;18758;14316;15797;13078;13829;2528;913;21480;2376;6331;17544;13849;1195;19063;14820;1978;21343;6651;3126;19922;22410;8707;5735;273;1490;4250;11635;13073;542;16458;11118;14330;19546;13343;1862;20780;7633;16038;3633;9110;2628;6998;1128;15445;14845;18611;11960;16524;7357;21569;9395;1899;16287;13150;14417;6851;7598;19306;18322;20431
KEGG_CHEMOKINE_SIGNALING_PATHWAY	19740;14498;2002;12531;5622;8453;14134;3553;8740;21803;9217;14588;15293;12665;12665;21428;4458;19099;17187;19321;3485;14986;3430;21792;2703;15977;15570;17483;14863;1842;21274;21198;18669;18828;8379;11307;8005;1178;751;17453;12932;19478;10780;11130;9232;10908;9897;12921;2539;2259;18959;21087;5285;17977;10028;22008;18255;15797;21235;22517;445;11925;13829;12412;19203;1469;2376;18142;534;20055;1873;14820;21343;3126;2462;20909;22410;6700;8707;1490;17571;10119;9028;14354;13073;6850;16458;20986;14959;19983;15218;2440;15538;7530;6851;5697;569
KEGG_PHOSPHATIDYLINOSITOL_SIGNALING_SYSTEM	12542;7993;22517;445;13861;16542;14246;3054;15669;3125;2512;21353;3148;15037;15653;505;12421;14660;14103;14863;2681;19994;4669;21112;19125;8228;18979;17762;2893;581;10935;2341;3804;16847;11929;15218;15538;7530;4399;5941;20355;14734
KEGG_NEUROACTIVE_LIGAND_RECEPTOR_INTERACTION	3894;22009;18837;18009;18635;8516;11855;20278;8203;863;1652;4710;9026;9796;6353;269;16919;8785;14426;22324;12519;5052;19168;16512;20302;19373;5919;16985;10851;16152;9491;5665;296;10641;9921;1813;10393;7418;3367;7526;17583;5341;9012;14719;6785;3534;10362;3510;20983;4418;4486;1134;19284;7399;2786;5543;15595;20590;20396;19824;16821;3343;12044;7580;5241;2700;16426;3428;10583;5799;22097;18681;4331;16800;16235;6673;6337;20188;2758;3993;1987;8437;1279;18972;17540;2331;491;10466;15639;11061;11420;637;2901;11844;5060;19914;18008;3614;10451;11514;3242;5816;17459;6301;15048;9000;8763;22564;14075;3484;18952;20728;885;3438;1889;12041;7102;16075;10880;13485;11789;18874;15214;14540;8012;6198;14619;14374;11258;3403;14286;8510;12965;7978;15802;22130;4715;14785;14273;12018;6372;15742;17264;17006;124;12971;14210;6778;13871;18020;3454;6923;412;17280;17438;10890;14551;19232;12255;11190;16586;16130;6523;16511;20582;11884;1296;482;15355;18622;17018
KEGG_CELL_CYCLE	19126;9626;12990;5373;11830;16499;11310;3127;11453;15115;2490;4585;9423;11142;17955;7602;2749;19315;6838;C8747.9;18429;2221;13123;6173;17078;9099;11700;19910;861;19468;3344;21982;21827;2837;6173;7852;2106;4909;9124;13654;9451;19931;19893;11741;1114;9366;669;6691;14354;21531;9655;21205;2670;6932;18133;18156;13872;2068;11989;101;12492;648;13988;8967;11744;7820;997;5322;3390;10676;14792;11780
KEGG_OOCYTE_MEIOSIS	19126;1207;1668;16568;11830;2512;11089;3127;15115;14141;18828;12932;2893;9423;11142;18761;19315;11809;194;18429;2446;18959;12354;15354;11929;6173;17078;11700;20355;3344;21982;21827;6173;4909;9124;19931;1873;14930;11807;19111;669;6691;19994;4669;8500;4000;18133;7021;6780;6491;13089;11744;3628;5322;3468;14734;7223
KEGG_P53_SIGNALING_PATHWAY	21963;4909;5373;13787;18305;7851;21892;11046;12738;110;11310;6410;C10565.5;19275;22080;10853;7395;18118;21531;2490;16038;20907;1375;5642;17793;3804;13123;17028;11989;6795;13150;11744;9419;21982;1313;21827;14792
KEGG_UBIQUITIN_MEDIATED_PROTEOLYSIS	19126;7733;20939;19708;9450;186;6271;11830;1479;21976;21944;C11046.4;11310;237;22080;20642;3127;14141;11425;11826;10184;14252;9132;9271;11142;20907;21263;5642;16808;12380;9133;10801;14850;21581;12089;8592;17685;17078;7643;16056;21302;9124;13787;11917;7171;19931;12507;14364;8774;13501;19893;16621;45;6691;17209;3472;6640;22390;7259
KEGG_SNARE_INTERACTIONS_IN_VESICULAR_TRANSPORT	3263;16853;15087;14636;21655;15343;12472;5544;963;15446;7465;5869;21279;17310;4235;20744;22395;2314;4994;15969
KEGG_REGULATION_OF_AUTOPHAGY	8432;2528;913;20389;14330;10048;7108;581;19063;6651;19922;5735;1761;10305
KEGG_LYSOSOME	11426;15601;5846;18393;547;7662;5127;12462;17472;13762;10920;10714;16221;4203;3281;5116;19288;1405;3403;13747;3314;16639;805;14179;4313;15611;16131;14649;13466;12068;15103;8808;3712;20873;10050;20053;3463;14651;19859;13569;6656;5801;17515;1708;2242;3249;13542;4695;12539;5293;3391;11220;1383;5496;5977;10709;20227;9254;3101;578;19767;20413;10220;10381;17631;18731;2033;17885;7717;4591;2071;21028
KEGG_ENDOCYTOSIS	6301;22568;8453;8516;11855;13028;16568;18185;2602;6873;11310;11017;10785;15037;21477;15653;19099;10272;9639;7618;13679;8321;3634;7745;14252;22336;9699;17747;9545;20776;12312;10814;13268;8592;21348;372;19608;19686;9682;7053;22553;11917;1469;16241;12403;18276;2529;6058;15707;21520;1028;22244;12486;7618;1195;10662;11000;8473;3126;20909;12804;19606;19380;6589;17209;10119;11720;542;6362;6579;17629;15445;16570;19420;21137;15204;19983;17885;17405;7717;17610;5697;5695;13974
KEGG_PEROXISOME	6220;8478;13272;17938;6298;5566;11279;21907;8725;8587;16560;3077;2090;823;5710;3711;156;22482;16751;21147;4472;6292;20503;3533;13036;3595;8640;7924;13497;20885;11167;4799;16787;4449;15391;9924;176;1658;8038;4635;4552;16278;19086;13602;19216;20499;13237;1157
KEGG_MTOR_SIGNALING_PATHWAY	21963;1207;22399;22252;5870;12499;9217;10632;4458;14930;1854;21517;6794;14863;1755;16422;10048;21326;11758;15218;15538;7530

KEGG_APOPTOSIS	10477;18255;11491;13078;5189;19708;1668;16931;19526;2013;5848;17210;11046;12738;20533;6225;6410;4458;11089;6561;15977;15570;16186;14863;9028;1842;18118;18828;4226;11118;1862;20323;16038;3633;20379;13784;9110;7832;14008;14297;18959;15218;15538;882;13150;7530;2928;16626;2
KEGG_CARDIAC_MUSCLE_CONTRACTION	10456;18233;13860;3654;4303;5386;17428;21023;21354;16653;7046;19366;9754;11098;11354;6658;13361;15741;12636;11023;22401;5554;21637;15176;8336;15590;8687;11590;21552;13634;8462;9434;20586;13486;16579;18278;2464;239;8742;11658;14482;18951
KEGG_VASCULAR_SMOOTH_MUSCLE_CONTRACTION	21022;2125;4336;7570;9217;2512;21022;2637;18828;2893;1637;12932;18959;11929;15799;14633;4892;167;21235;22517;7993;445;10339;9561;15669;18142;14674;10926;22401;C10773.4;13427;14717;15377;1873;17004;21637;15176;11807;13282;18627;19305;3343;4102;21862;15167;16426;19994;4669;1924;8437;1279;3913;14959;10466;15639;11061;10328;14734
KEGG_WNT_SIGNALING_PATHWAY	14573;807;9399;15774;15777;1668;18425;8858;3957;8791;18832;16499;10694;7374;8119;21090;10340;12157;14559;11089;3478;18810;8554;14141;18828;11142;5642;5342;16083;1731;194;2446;18959;7605;5142;22517;7993;445;12330;13981;15669;10090;1748;6073;16448;22172;18203;7424;12264;6480;11741;1114;C9571.7;11352;4925;14354;9285;17527;811;2289;18441;4000;7021;6491;16246;14959;99;18795;2484;3628;3468;248;7223
KEGG_DORSO_VENTRAL_AXIS_FORMATION	16306;15023;9114;1873;6700;5752;19111;14802;469;17571;15044
KEGG_NOTCH_SIGNALING_PATHWAY	18879;16306;15023;12586;19445;22347;13151;18896;16697;21101;16499;20166;2380;17585;6073;16448;1070;12264;13559;13587;2144;8736;3390;10676;228;15253
KEGG_HEDGEHOG_SIGNALING_PATHWAY	21544;10135;15049;11542;8119;2058;14559;4200;6480;4925;16863;4307;15093;3616;14354;9285;14141;17527;811;2289;18828;18441;16083;14384;9807;18959;18384;7605;2777
KEGG_TGF_BETA_SIGNALING_PATHWAY	18146;13078;10428;4626;9042;16499;5693;21517;11741;21516;11453;1114;2009;17209;3616;6790;14969;14330;4000;7021;13119;18125;11142;19720;12953;6491;1378;2628;2425;14679;9572;3259;6940;11300;8739;17955;14959;6748;20927;18384;18201;2777;1602;7364;20214
KEGG_AXON_GUIDANCE	15531;1668;732;20786;8107;14287;13312;2797;11596;11089;20938;2264;8055;8554;14485;15650;1018;8005;665;12182;3851;4292;21087;14299;15336;16732;5142;10030;10671;10006;5559;18294;12648;1469;6160;9659;10886;17208;22172;18997;20580;6642;7587;6473;12174;8855;10119;14354;7773;16883;13291;14959;20917;11875;22110;9666;8141;20328;18256;7949;7223
KEGG_VEGF_SIGNALING_PATHWAY	21235;7993;1668;5680;10339;7544;5848;15669;21022;4336;7570;9739;319;10926;C10773.4;22172;14717;10577;15377;1873;1195;21022;4458;11089;7033;14863;10119;8554;13384;4388;9897;15218;15538;7530;4399;5941;15799;14633;5142;7223
KEGG_FOCAL_ADHESION	14573;19708;4626;16568;12499;5569;14748;4458;19164;12934;12152;4218;3202;5308;21118;16809;14863;16186;2464;7936;13381;22413;13777;15486;1602;20214;14131;3808;3324;9561;5848;860;10090;1748;1873;16271;6700;11807;892;10119;5666;14959;3804;17999;15098;895;11922;5346;22529;13207;910;1267;15688;6865;14391;15691;9217;21570;15293;18948;14992;21301;1836;271;C14348.5;12637;16422;9897;6034;14297;6847;2259;21087;21326;17977;8192;6978;10006;21235;7993;15669;22172;1195;17004;17571;11352;14354;1972;542;4305;12517;777;15218;15538;7530;18256
KEGG_ECM_RECEPTOR_INTERACTION	5346;22529;14131;17995;21505;3808;4626;13207;21853;3324;910;1267;15688;15691;21570;14992;1836;19164;271;12152;C14348.5;4218;3202;5308;21118;16809;892;12893;5666;1972;14592;4305;12517;777;17999;415;22413;13777;15486;12527;11922;1602;20214
KEGG_CELL_ADHESION_MOLECULES_CAMS	1867;4005;455;16290;20510;11901;3817;11361;7135;11505;12790;10736;2871;16490;4045;11791;1836;271;5946;15035;13812;18995;9038;8267;12876;21124;21550;3232;1080;14437;C11652.5;2752;6645;14671;12557;2785;10930;22072;20913;5270;20378;9510;14597;21857;14631;11114;6428;18652;10080;10436;21155;20783;21136;5830;6174;7466;7498;5666;17663;21194;1494;16239;8985;12517;12785;11671;16004;13332
KEGG_ADHERENS_JUNCTION	3953;12412;15437;16568;16499;860;5023;5055;20055;22172;4607;12157;11741;C9571.7;1114;16758;933;7498;12440;16239;1378;9572;11531;1731;18795;17606;19744;6008;18256;8192;20378;6978;5270
KEGG_TIGHT_JUNCTION	3953;19080;18354;16625;22053;18041;2555;11302;6734;5569;4458;12934;933;13812;8267;16282;6645;5448;8005;2464;7936;13381;7552;10930;3969;20074;22072;8192;6978;7993;14597;11114;6428;16241;860;15669;18142;14674;21155;13427;20909;10119;12440;6362;13265;19948;21627;17542;3523;4000;7021;6491;19967;8985;3804;11562;11989;13332;16481
KEGG_GAP_JUNCTION	19589;21235;7993;22517;445;4909;3534;15669;6865;2512;1873;13522;15214;6700;13282;19305;17571;14433;11497;10119;4307;19994;4669;12637;886;542;18828;8005;22473;11497;4331;16800;12932;21078;6034;18959;22166;414
KEGG_COMPLEMENT_AND_COAGULATION_CASCADES	6301;14131;8269;C10884.2;20822;16057;17169;12627;2417;253;13530;13131;7256;14127;1886;12684;14737;2527;7209;22475;5440;20302;16043;7090;5919;19733;11662;18162;8188;9921;1942;16325;12708;4429;2181
KEGG_ANTIGEN_PROCESSING_AND_PRESENTATION	9510;8175;13913;14441;12423;2528;913;17472;13762;2529;6058;17855;5029;767;4203;19063;20016;6651;19922;5735;19606;12876;4517;9400;19754;6840;C11652.5;3429;19441;15504;12557;1815
KEGG_RENIN_ANGIOTENSIN_SYSTEM	7704;7909;7697;3403;6453;13948;7707;1813;8560;8794;17459
KEGG_TOLL_LIKE_RECEPTOR_SIGNALING_PATHWAY	14573;20510;14498;7661;2013;18154;13696;12157;4458;5116;C14348.5;7333;15977;15570;14863;17096;18625;1842;14252;10759;19478;4596;17928;18255;13078;2528;913;16931;17210;10090;17544;1748;9739;10577;1873;11290;3699;19063;6651;19922;5735;15010;9028;11352;13343;7633;9110;7832;11671;15218;2440;15538;7530;13582;13783;20461;11499;10189;20431
KEGG_NOD_LIKE_RECEPTOR_SIGNALING_PATHWAY	18255;13270;15797;13078;19708;5622;3553;9200;8740;21803;4900;10090;1748;9739;21888;10577;2366;1111;12157;9333;18915;15977;15570;9028;19534;11352;5370;1842;15291;14252;4596;10423;9110;2413;19670;2647;20431
KEGG_RIG_I_LIKE_RECEPTOR_SIGNALING_PATHWAY	18255;13078;2528;913;20389;16931;2013;10090;1748;17544;9739;21944;10577;12157;19063;6651;19922;5735;7333;15977;15570;15010;3666;9028;11352;1842;1597;14252;10759;13343;14008;13401;21386;13233;16524;2359;18749;14417;19359;545
KEGG_CYTOSOLIC_DNA_SENSING_PATHWAY	18255;2528;913;2013;9333;19063;6651;19922;5735;4017;7333;1809;15977;15570;15010;9028;14574;5370;1842;16681;13343;16924;4579;9110;13401;13233;18749;20431
KEGG_JAK_STAT_SIGNALING_PATHWAY	19536;14498;2002;6958;16499;11659;21587;6873;4035;4458;21428;2703;17057;1524;14863;10184;4226;14774;21263;2552;21581;8592;19852;15943;14316;12856;2528;913;281;14859;19097;5023;6331;17544;13849;19063;6651;19922;6700;5735;273;17987;17571;11635;10422;22445;14829;14330;19546;13343;7633;15445;1128;6998;18611;11960;7219;16524;20900;7357;21569;9395;15218;15538;16287;13041;7530;10451;14417;13843;7598;19306;219
KEGG_HEMATOPOIETIC_CELL_LINEAGE	14316;13078;21505;11361;7135;21853;278;18239;15172;6331;21796;20194;22244;11791;7618;20533;11635;1524;9639;12876;13571;7618;5636;C11652.5;14592;19625;8210;9110;15445;12517;777;7357;2785;12754;19241;9905;136;18548;10719;16024;7598;19306;18322;20431
KEGG_NATURAL_KILLER_CELL_MEDIATED_CYTOTOXICITY	C12233.8;5189;1668;14326;9217;15293;5204;11089;9489;21640;1741;1524;14863;5020;9446;15992;8554;21020;21087;18039;4399;5941;5142;22008;7387;8175;21235;13913;7993;14441;13078;21857;2528;913;18652;5023;15669;11046;22172;17855;1873;6410;19063;6651;14187;19922;5735;6700;17571;10119;15326;11118;14330;19546;13343;7231;1862;1924;7633;16038;16063;3633;6628;15218;15538;13150;7530;18256;7223
KEGG_T_CELL_RECEPTOR_SIGNALING_PATHWAY	C12233.8;14573;3817;1668;2871;15293;13696;12157;4458;11089;15977;15570;9038;8055;21504;14863;1524;8554;1842;C11652.5;21020;3851;20379;12754;21087;19241;8592;18039;4399;5142;7387;6879;10006;18255;21235;13078;5023;10436;10090;9739;13427;10577;1873;11290;6700;7466;17571;10119;9028;20800;14354;6850;14330;12785;11989;15218;15538;7530;2201;18256;7598;7223
KEGG_B_CELL_RECEPTOR_SIGNALING_PATHWAY	14573;18255;21235;1668;5023;3125;5682;22172;21796;1873;15293;11791;8919;9480;4458;7100;11089;6700;15977;15570;17571;21504;14863;9028;10119;9859;8554;1842;14354;7231;4268;10567;15218;15538;7530;5941;2201;5142;19236;7223
KEGG_FC_EPSILON_RI_SIGNALING_PATHWAY	21235;7993;13078;10339;14326;20364;3307;18142;14674;3125;10090;21022;1748;4336;7570;9739;10926;22172;C10773.4;10577;14717;15377;1873;15293;11290;21022;3699;4458;6700;17571;14863;1524;10119;11352;7231;4268;15218;15538;18039;7530;4399;5941;15799;14633;18256;7598;19236
KEGG_FC_GAMMA_R_MEDIATED_PHAGOCYTOSIS	7544;7570;15293;4607;21022;18948;15037;4458;15653;8266;9136;4645;1741;14863;1018;13268;21087;17977;4399;5941;19686;9682;21235;7993;12412;15669;18142;3125;14674;20055;C10773.4;15707;1873;8473;2881;21517;12804;7466;18197;4388;17495;7773;7231;15218;6981;15538;3766;7530;192
KEGG_LEUKOCYTE_TRANSENDOTHELIAL_MIGRATION	1867;3953;11361;17092;9702;5569;10201;12495;8843;4045;15293;18948;5946;12934;358;933;13812;8267;14863;6645;8005;2464;7936;9897;13381;6847;2259;10930;4399;5941;22008;C11622.6;22072;8192;6978;7993;14597;11114;6428;18652;1469;860;15669;17458;21155;9739;10577;21136;6850;12440;8985;14959;13332;15218;15538;7530;3739
KEGG_INTESTINAL_IMMUNE_NETWORK_FOR_IGA_PRODUCTION	12876;7380;9510;20510;3817;4149;11925;22445;14829;12790;2871;9232;20379;1469;10908;1367;2376;12785;11671;12557;1836;5285;7598;20431
KEGG_CIRCADIAN_RHYTHM_MAMMAL	6287;20283;4307;16167;20834;20592
KEGG_LONG_TERM_POTENTIATION	21235;22517;7993;445;1207;1668;16499;9561;15669;17280;17438;9217;14551;2512;1873;4486;14930;11089;11807;10119;19994;4669;18828;2893;1924;4544;194;2446;18959;11929;14734;7223
KEGG_NEUROTROPHIN_SIGNALING_PATHWAY	14573;8441;1207;21892;18485;21944;9217;4458;15977;15570;21504;14863;185;1842;14252;2893;20323;14297;19315;4544;194;2539;2446;15085;20053;11929;4794;17977;4399;5941;3344;9243;21235;5848;17210;18142;10090;1748;9739;10577;1873;12738;11290;13522;14930;16271;6700;17571;10119;11352;14354;13384;12299;20135;13784;7832;15218;15538;7530;20311;2059;21111;14734
KEGG_LONG_TERM_DEPRESSION	21235;7993;22517;445;14075;8240;16568;10339;15669;21022;4336;7570;10926;9217;C10773.4;2512;14717;15377;1873;20273;4486;21022;13282;19305;14433;10119;19994;4669;8005;4000;1924;7021;6491;4091;1803;15799;14633;19236
KEGG_OLFACTORY_TRANSDUCTION	16281;20183;19099;20367;17115;15835;3554;6263;194;2446;15827;11929;13362;2672;6442;20997;13671;20023;1432;16314;12694;18981;2356;15128;3362;3143;1712;16614;9575;3493;12753;8193;18404;6049;5316;1966;11833;6675;13222;4831;10575;11871;14014;17322;4782;17882;14734;2575;2478;10575;4191;10313;18828;15735;2338;6593;7727;10016;6907;21686;15147;15964;19764;9912;15090;11443;15910;13767;12932;2893;12755;19837;17248;17186;9564;18959;7505;14686;1624;3022;1269;8551;20068;19305;14433;18221;6643;11900;6675;18128;1650
KEGG_TASTE_TRANSDUCTION	18939;124;10868;11049;4192;2512;10623;3430;8454;9139;18704;10283;9597;21274;18828;11307;5943;15320;8102;3503;9035;18959;2571;9308;13726;19239;2551;6399;14045;20360;15816;1829
KEGG_REGULATION_OF_ACTIN_CYTOSKELETON	19925;2555;5569;8266;6016;12934;21792;9136;1801;14863;14751;1018;2464;7936;5919;13381;16847;20074;21845;2747;1209;11339;12412;18383;19188;22250;9561;22509;860;1873;10662;19712;12613;8898;11807;18427;892;10119;5666;11881;7773;7252;14959;13089;11975;15204;8602;5875;6301;11361;7407;8858;910;6865;9217;12588;15293;4607;21301;14992;15037;1836;15653;271;6911;3261;12637;8510;7978;15802;9897;10965;6034;15084;2259;21087;17977;8192;6978;10006;21235;20055;19232;22172;17004;17571;18197;542;3523;1924;20986;12517;777;15218;15538;7530
KEGG_INSULIN_SIGNALING_PATHWAY	19460;5870;16372;434;9217;4458;13933;1961;6365;505;21646;14863;18962;13510;18862;10184;18828;4234;2893;10048;13565;21263;8281;18959;21797;15085;8592;11929;19427;4794;17977;4369;2928;19744;21235;11491;21963;7762;22399;14859;9794;15159;5848;13876;16241;9901;3125;10090;1748;1873;17667;10632;16174;16271;20035;21517;20909;6700;11807;14852;16758;1878;6794;17571;10119;11352;14354;8368;6135;1924;15225;16268;15

KEGG_GNRH_SIGNALING_PATHWAY	14573;21022;7570;4336;9702;21944;2512;10344;21022;6353;18828;2893;12932;194;2446;18959;12018;5392;11929;15799;14633;19686;9682;446;22008;414;21235;22517;7993;445;10339;18142;10090;1748;9739;10926;22401;C10773.4;14717;10577;15377;1873;11290;21637;3699;15176;6700;17571;10119;11352;19994;4669;11420;19914;14734
KEGG_PROGESTERONE_MEDIATED_OOCYTE_MATURATION	19126;21235;1207;4909;9124;16568;11830;19931;10090;1748;9739;9217;10577;1873;4458;14930;19111;3127;6691;669;14863;18962;11352;18828;8005;2670;1924;12932;9423;13565;13089;11809;C8747.9;18429;18959;15218;17078;15538;9099;7530;11700;5322;19910;21982;14792
KEGG_MELANOGENESIS	13415;3957;8791;16499;10694;16891;8119;14559;9179;7618;18828;8005;12932;2893;10309;16083;194;2446;18959;7605;11929;21235;22517;7993;445;7182;20820;278;15669;3534;6073;16448;1873;7618;12264;16001;6480;C9571.7;10119;4925;14354;9285;17527;811;2289;18441;99;18795;248;14734
KEGG_ADIPOCYTOKINE_SIGNALING_PATHWAY	18255;4725;13078;7762;21853;10825;22399;8864;16891;10090;1748;13427;3935;10545;17667;15451;4458;6365;15977;15570;21504;9028;11352;6508;1920;1842;1755;4234;10048;21263;14008;14508;21797;15085;13602;1607;4369;4042;2059;1157
KEGG_TYPE_II_DIABETES_MELLITUS	14863;19460;11352;13078;7762;10184;1050;22399;14859;21263;18142;14674;10090;434;1748;22401;5486;14508;15085;2692;15218;15538;16304;20909;7530;16122;14852;16758;2059
KEGG_TYPE_I_DIABETES_MELLITUS	12876;9400;9510;20510;3817;13078;14330;9110;14925;17544;12557;22302;653;20533;5204;13150;9489
KEGG_MATURITY_ONSET_DIABETES_OF_THE_YOUNG	11367;19460;6465;12586;1050;18592;14922;20552;14937;19426;15372;7382;10409;5486;1728;14852
KEGG_ALDOSTERONE_REGULATED_SODIUM_REABSORPTION	14863;12745;7993;10200;21023;5943;7715;9754;11098;15669;10537;2870;15085;8336;8687;15218;15538;7530;10133;16758;2059
KEGG_VASOPRESSIN_REGULATED_WATER_REABSORPTION	19919;12472;7182;18828;14428;13415;12932;10309;22466;7458;4901;9545;18959;19076;4983;1147;1799;18032;7649;9243;19380
KEGG_PROXIMAL_TUBULE_BICARBONATE_RECLAMATION	14033;19981;16517;21023;19365;12924;8336;8687;5558;9754;2885;11098;3505
KEGG_ALZHEIMERS_DISEASE	3654;21231;4634;1668;7967;13919;15741;5554;2512;8496;15741;15741;21216;11089;11918;16472;17494;16186;13120;18118;18278;18896;2893;3321;1416;2492;4031;11658;8699;11967;22386;11929;11755;10966;228;18233;4303;10477;22517;445;13078;16931;7960;9578;5848;11354;6658;13361;13256;17280;11023;17438;11046;22401;14551;20273;21637;15176;6410;11590;8462;9434;20586;20911;3329;20703;19994;22293;4669;14354;19445;15297;17520;12482;304;16579;239;8742;9110;12216;177;15466;6498;13016;3740;5250;1159;17435;6837;7096;22368;8141;18951;13150;14734;7223
KEGG_PARKINSONS_DISEASE	3654;9450;13919;15741;5554;15741;19256;15741;21216;11918;16472;17494;9671;18118;12926;18278;9132;1416;4031;11658;14850;6153;8327;10966;16044;18587;18233;4303;7960;9578;11354;6658;13361;13256;11023;7302;13501;6410;11590;8462;9434;20586;20911;3329;20703;22293;15297;17520;12482;304;16579;239;8742;12216;177;15466;6498;13016;3740;5250;1159;17435;6837;7096;22368;1085;18951;12146
KEGG_AMYOTROPHIC_LATERAL_SCLEROSIS_ALS	13078;6298;1668;14451;5848;6154;18485;17280;823;17438;9739;11046;14551;10577;12738;20273;7494;4486;3699;9333;6410;6756;11089;18118;1994;14297;10353;1439;7223
KEGG_HUNTINGTONS_DISEASE	12689;11459;3654;20146;12705;17172;11683;18341;13415;16499;13919;10820;13889;15741;5554;15741;15741;4983;21216;19423;11918;16472;17494;9874;18118;12926;18278;1416;10309;4031;11658;6153;20927;8327;10966;9370;1147;10827;21220;14439;6125;5532;18587;18233;4303;22517;445;6257;7182;7960;9578;18191;8452;11354;6658;13361;13256;11023;17438;823;5710;12738;6410;11590;18032;8462;9434;20586;20911;21985;3329;9868;20703;19994;22293;15297;17520;12482;304;16579;239;8742;1994;17370;12216;15466;6498;3740;13016;5250;1159;17435;7096;6837;22368;16366;18951
KEGG_PRION_DISEASES	10484;17663;18828;16043;9110;823;1873;12738;18959;20533;17537;1942;3202;18256;1886;20431;14737;2527;7209
KEGG_VIBRIO_CHOLERAE_INFECTION	15677;7993;6801;17829;12462;1834;15669;19421;14731;16221;3249;6059;12539;18774;18828;12932;5365;1274;20873;18959;9225;15482;4399;5941;6654;6583;9900
KEGG_EPITHELIAL_CELL_SIGNALING_IN_HELICOBACTER_PYLORI_INFECTION	14573;15677;18255;15797;17829;12462;1834;10090;1748;9739;10577;14731;16221;6410;3249;12539;15977;15570;13812;9028;11352;1842;20379;5365;1274;2492;20873;21087;15204;9225;15482;4399;5941;446;6654;6583;19236;9900
KEGG_PATHOGENIC_ESCHERICHIA_COLI_INFECTION	19589;7993;12412;3534;6734;20055;18154;8266;11497;8055;17096;886;11497;3851;14959;21078;11562;11187;13783;22166;18256;22072;5270
KEGG_LEISHMANIA_INFECTION	14573;9510;14498;13078;11361;17210;5023;17544;9739;10201;8843;3711;10577;12157;20533;15977;4645;15570;1741;12876;1842;14252;14330;19546;4596;9110;7832;17955;12557;13783;7598;11499
KEGG_PATHWAYS_IN_CANCER	14573;14498;19708;8412;21544;16568;9608;3957;8791;10694;19492;12499;8119;14559;4458;19164;4200;12152;5859;4218;3202;18810;2703;5308;13817;21118;15977;16809;15570;933;14863;9639;16863;19853;14252;16981;10759;11142;1378;9572;17955;2605;8281;21581;13777;8592;7605;4399;5941;19686;12641;2747;1209;11339;18383;22399;19188;278;10135;22250;22509;5848;10090;1748;3711;12738;1873;7618;6410;19712;12613;8898;6700;18427;6480;11741;14802;1114;9028;10119;4925;20292;9285;17527;811;12440;11881;2289;18441;2670;7252;99;3804;18795;11253;8858;16499;15688;6865;9702;14908;12495;15691;9217;15551;21570;8400;11310;3742;271;5253;7618;1842;9193;16309;16422;16083;6034;14297;9636;1585;21326;4702;17977;10030;21827;5270;7852;18255;21235;7227;7993;10825;16931;10860;15669;7171;6073;16448;11046;22172;3935;10545;12264;19893;C9571.7;17571;11352;4869;14354;21531;542;4305;1924;6780;12517;777;14384;11989;16366;2484;101;12492;15218;15538;13150;11744;7530;3390;248;10676;2043
KEGG_COLORECTAL_CANCER	14573;21235;8858;19492;5848;10090;1748;9217;22172;15551;12738;1873;4458;6410;18810;11741;1114;C9571.7;14863;11352;9193;14354;1924;1378;9572;17955;14297;18795;9636;4702;15218;15538;7530;10030
KEGG_RENAL_CELL_CARCINOMA	14573;10006;21235;10825;16499;7171;12499;9217;4829;1873;4458;3742;16271;6700;14802;17571;14863;10119;4869;16309;1924;16422;11142;17955;21087;21326;15218;15538;7530;17977
KEGG_PANCREATIC_CANCER	7852;18255;21235;14498;10860;5848;12499;10090;1748;14908;22172;9217;1873;4458;11741;1114;15977;14863;9028;11352;1842;21531;542;1924;16422;1378;9572;17955;2605;11989;1585;21326;101;12492;15218;15538;7530;19686
KEGG_ENDOMETRIAL_CANCER	21235;8858;5848;9217;15551;1873;4458;6700;18810;C9571.7;933;17571;14863;10119;14354;12440;542;1924;3804;18795;2539;15218;15538;7530;527
KEGG_GLIOMA	7852;21235;7993;22399;16568;15669;6865;9217;1873;11310;4458;6700;17571;14863;10119;21531;542;1924;2893;3804;6034;194;2446;11989;101;12492;11929;15218;15538;7530;4399;5941;14734
KEGG_PROSTATE_CANCER	7852;18255;21235;7182;22399;13415;11253;16568;9608;16499;5848;6865;9217;1873;11310;4458;6700;18032;C9571.7;15977;15570;17571;14863;9028;10119;1842;14354;12637;542;1924;6780;10309;5630;3804;6034;14297;8281;18795;101;12492;15218;15538;11744;7530;21827
KEGG_THYROID_CANCER	12641;10119;19853;9217;18795;1873;3935;10545;16366;5859;C9571.7;5270
KEGG_BASAL_CELL_CARCINOMA	21544;8858;3957;10135;8791;10694;6073;8119;16448;14559;12264;4200;18810;6480;C9571.7;16863;4925;14354;9285;17527;811;2289;18441;99;16083;14384;18795;7605;248
KEGG_MELANOMA	7852;21235;2747;1209;11339;18383;16568;19188;22250;22509;5848;6865;9217;1873;11310;4458;19712;12613;8898;18427;14863;10119;12637;21531;542;11881;7252;1924;3804;6034;11989;101;12492;15218;15538;7530;5270
KEGG_BLADDER_CANCER	10119;7852;21235;21531;542;11648;1924;16422;12499;9702;9217;12495;1873;11310;11989;21326;101;12492;5253;5270
KEGG_CHRONIC_MYELOID_LEUKEMIA	7852;18255;21235;5848;9217;1873;11310;4458;6700;11741;2703;1114;15977;15570;17571;14863;9028;10119;1842;21531;1924;1378;9572;17955;11989;8592;101;12492;15218;15538;7530;17977;3390;10676
KEGG_ACUTE_MYELOID_LEUKEMIA	18255;7227;21235;8412;22399;5870;5848;9217;1873;8400;7618;4458;21517;6700;2703;C9571.7;15977;17571;14863;9028;10119;7618;20292;1842;2670;1924;18795;2484;15218;15538;13041;7530
KEGG_SMALL_CELL_LUNG_CANCER	7852;18255;19708;15688;15691;3711;21570;3935;10545;19893;4458;271;19164;12152;4218;3202;5308;21118;16809;15977;15570;14863;9028;1842;18118;4305;14252;16981;10759;12905;12517;777;3804;14297;11989;21581;13777;101;12492;15218;15538;11744;7530;16153;21827
KEGG_NON_SMALL_CELL_LUNG_CANC	7852;21235;7993;5848;15669;9217;1873;3935;10545;4458;6700;17571;14863;10119;21531;542;12905;1924;2539;11989;101;12492;15218;15538;7530;
KEGG_ASTHMA	12876;9510;13078;8740;14326;10984;20364;3307;12785;11671;12557;17183;7598
KEGG_AUTOIMMUNE_THYROID_DISEA	9510;20510;3817;2528;913;5341;2485;6353;19063;5204;6651;19922;5735;9489;9038;12876;12785;11671;12557;13150;7598
KEGG_SYSTEMIC_LUPUS_ERYTHEMATOSUS	8829;20510;3817;14495;15345;21338;12260;14359;21338;19118;3403;4871;4645;1886;10124;14737;1741;2527;7209;12876;16043;18167;4871;4871;12557;6913;4871;14587;8188;1942;14587;12708;8192;6978;9510;21338;13078;4871;4871;21114;4871;4871;4871;4871;4871;860;4871;4871;17280;17438;21338;21338;21338;21338;21338;21338;4871;21338;21338;21338;21338;4308;2978;5891;13065;6097;14330;2978;11570;11584;2978;18449;2978;2978;12785;11671;782;21338;19118;12260;2888;2620
KEGG_ALLOGRAFT_REJECTION	12876;9510;20510;3817;13078;14330;12785;11671;17544;12557;5204;13150;9489;7598
KEGG_GRAFT_VERSUS_HOST_DISEASE	12876;9510;13913;20510;3817;13078;14330;9110;12557;17855;20533;5204;13150;9489;20431
KEGG_PRIMARY_IMMUNODEFICIENCY	C12233.8;7380;9028;19754;12423;6840;C11652.5;2871;6331;4661;12785;11671;4268;21796;1428;16017;12754;5029;12089;9480;21428;7387;7466
KEGG_HYPERTROPHIC_CARDIOMYOPATHY_HCM	10456;13860;5386;17428;13078;16653;21354;7046;2821;19366;910;16630;12636;22401;21637;15176;17667;15590;14992;1836;271;13634;14171;11611;892;5860;17901;17931;5666;13486;4305;2464;4234;10048;19786;12517;17955;777;14482;4369;3801;7707;20431
KEGG_ARRHYTHMOGENIC_RIGHT_VENTRICULAR_CARDIOMYOPATHY_ARVC	10456;13860;5386;17428;21354;16653;8412;2821;7046;19366;910;16630;20109;860;9579;12636;22401;21637;15176;14992;1836;271;C9571.7;13634;4657;933;18995;11611;892;5860;17901;17931;5666;12440;13486;4305;22473;12517;777;14482;18795;3801;8192;6978
KEGG_DILATED_CARDIOMYOPATHY	10456;13860;5386;17428;13078;21354;16653;2821;7046;19366;910;16630;12636;22401;21637;15176;15590;14992;1836;271;13634;14171;11611;892;5860;17901;17931;5666;13486;21979;18828;4305;2464;12932;19786;12517;17955;777;14482;18959;3801
KEGG_VIRAL_MYOCARDITIS	9510;20510;3817;3185;18652;22053;11302;11046;22172;6410;20765;5204;11611;5860;12876;17901;17931;4305;12785;11671;12557;895;3969;20074;1
BIOCARTA_RELA_PATHWAY	9028;18255;1842;13078;6189;14252;16931;16499;2013;15977;14008;15570
BIOCARTA_NO1_PATHWAY	18962;16085;11491;16653;12734;13565;21331;5919;319;10890;1195;1031;18959;14748;895;2928;19305;14433
BIOCARTA_CSK_PATHWAY	C12233.8;11491;12754;18959;19241;16499;21020;2928;7387;7466
BIOCARTA_SRCRPTP_PATHWAY	9099;11700;19910;6700;7993;21982;5122;4909
BIOCARTA_AMI_PATHWAY	6301;15691;12660;21570;5440;18162;8269;795;15688
BIOCARTA_GRANULOCYTES_PATHWA	4045;13078;20533;11361;18652;14330;15035;16043
BIOCARTA_LYM_PATHWAY	5946;4045;20533;7135;18652
BIOCARTA_ARAP_PATHWAY	13909;17462;3634;6801;20454;7717;12599;19988

BIOCARTA_AGR_PATHWAY	14573;10006;11352;16085;11559;15986;4305;9897;18091;6734;10890;21087;12264;11000;20927;7197;5308
BIOCARTA_AKAP95_PATHWAY	4369;2928;20274;11491;21982;18959;21232;8856;4909
BIOCARTA_AKT_PATHWAY	9028;18255;8281;2539;1842;15218;10451;3344;5848;15977;15570
BIOCARTA_ALK_PATHWAY	16911;4925;17723;18146;14354;14969;2464;20552;18125;20747;1378;9572;17807;17955;8739;12157;12264;18384;1114;2009
BIOCARTA_AT1R_PATHWAY	16911;14573;14031;10119;11352;21235;7993;21944;1873;21087;13948;6700;22008;17571
BIOCARTA_ACE2_PATHWAY	7704;15691;7909;12660;21570;7697;13948;7707;1813;15688
BIOCARTA_ASBCELL_PATHWAY	12785;11671;13150;3817;7598
BIOCARTA_DNAFRAGMENT_PATHWAY	882;10477;7957;9489;6561;10548;14222;6410
BIOCARTA_CHEMICAL_PATHWAY	14297;10477;11046;14498;7993;18118;12738;6225;6410;5848;9897;14755
BIOCARTA_SPPA_PATHWAY	10119;21235;6301;C10773.4;7993;1873;8005;7231
BIOCARTA_ATM_PATHWAY	14573;11352;C11046.4;12612;1842;13123;11310;2490;10860;21892;15977;15570;19447
BIOCARTA_AGPCR_PATHWAY	7993;11491;18959;2928;5697
BIOCARTA_BCELLSURVIVAL_PATHWA	10477;6410;8000;22399;6882;20764;15218;3795
BIOCARTA_BLYMPHOCYTE_PATHWAY	11671;7466;18652
BIOCARTA_BCR_PATHWAY	14573;10119;11352;8554;21235;7993;1668;7231;4268;21944;1873;15293;9480;7100;4399;6700;5142;19236;17571
BIOCARTA_BIOPEPTIDES_PATHWAY	10119;11352;19536;21235;7993;14498;2002;8005;194;2446;1873;8141;13948;4399;6700;1813;2703;18256;22008;17057;17571
BIOCARTA_NEUROTRANSMITTERS_PA THWAY	21838;4299;3749;13007
BIOCARTA_RANKL_PATHWAY	11352;10832;1842;14252;13343;20780;7633;15977;21035
BIOCARTA_CACAM_PATHWAY	7115;4544;194;2446;3992
BIOCARTA_CDMAC_PATHWAY	14573;10119;21235;7993;1842;1873;13078;4563;15977;15570
BIOCARTA_CARM_ER_PATHWAY	16911;15751;6189;6114;14405;14849;17370;16499;9906;14309;2380;21338;C11046.4;18260;13968;3390;10676;12180
BIOCARTA_CASPASE_PATHWAY	10477;18118;19708;6225;9333;2821;6410;18211;16220;5204;882;9489;9243;14755
BIOCARTA_CBL_PATHWAY	9639;7993;13679;542;6700
BIOCARTA_CCR3_PATHWAY	17458;10119;167;21235;7993;1873;14820;2464;7773;8740;14959
BIOCARTA_CD40_PATHWAY	12785;11671;9028;18255;14209;21944;1842;21589;14252;10759;20379;15977;15570
BIOCARTA_MCM_PATHWAY	7602;2749;14165;6838;2221;648;13988;8967;278;997;861;21827;2837
BIOCARTA_G1_PATHWAY	7852;14354;21531;4909;2670;17955;11989;19893;17737;19910;11741;3390;1114;21827
BIOCARTA_G2_PATHWAY	2713;C11046.4;13123;2068;11310;4909;21205;7643;9099;11700;19910;2490;9423;3344;21982
BIOCARTA_CELL2CELL_PATHWAY	4045;2259;12440;933;9897;8192;6978
BIOCARTA_LAIR_PATHWAY	13078;20533;18652;5946;15035;16043;20431;14737
BIOCARTA_CERAMIDE_PATHWAY	14297;11352;21235;21944;1842;12738;1873;1405;16931;2013;5848;15977;20935;14008
BIOCARTA_TID_PATHWAY	1842;13078;9574;16913;6672;14330;19546;15977;15570
BIOCARTA_CLASSIC_PATHWAY	8188;16043;1942;12708;1886;14737
BIOCARTA_COMP_PATHWAY	8188;16043;1942;16325;12708;20822;1886;4429;14737;19733
BIOCARTA_VDR_PATHWAY	2415;16499;3390;21963;18261;18260
BIOCARTA_HDAC_PATHWAY	16911;14031;7115;1668;16568;3699;15218;3344;5142;16758;3992
BIOCARTA_GCR_PATHWAY	319;17085;17723;1842;11190;1611;11855;15218;15977
BIOCARTA_CTCF_PATHWAY	3804;21531;11310;9480;7100;22399;15218;18125;1114;20747;1378;9572;2009;17955
BIOCARTA_CTL_PATHWAY	12754;19241;18652;5204;13150;21020;9489
BIOCARTA_CXCR4_PATHWAY	10119;21235;7993;1842;1873;2259;8005;15218;4399;1469;22008;15977;9897
BIOCARTA_CELLCYCLE_PATHWAY	7852;21531;11989;4909;21205;2670;7820;6932;19910;11453;21982;21827;11780
BIOCARTA_CFTR_PATHWAY	19421;6132;2928;11491;18959;11855
BIOCARTA_CYTOKINE_PATHWAY	17544;9400;8351;5370;13078;20533;14829;14330;13343;7598;20431
BIOCARTA_INFLAM_PATHWAY	1524;9400;13571;13078;14829;14330;13343;17955;17544;6034;7357;20533;7598;20431;11635
BIOCARTA_D4GDI_PATHWAY	14573;18118;6847;9333;6410;5204;9489;9243;14755
BIOCARTA_DC_PATHWAY	1524;17544;11671;2785;7661;19625;14330;6911;13343;13783;7598;11499
BIOCARTA_P35ALZHEIMERS_PATHWA	8141;8699;4307;10639;14354;16994
BIOCARTA_RNA_PATHWAY	18255;1842;20379;15977;21005;15570
BIOCARTA_MTA3_PATHWAY	19589;20538;15437;4756;11497;13320;3390;3712;5055;5270
BIOCARTA_SKP2E2F_PATHWAY	7643;2670;7852;21827;19893
BIOCARTA_CALCINEURIN_PATHWAY	8554;7993;20927;1668;2881;4399;5142
BIOCARTA_EGF_PATHWAY	14573;10119;11352;19536;21235;7993;14498;2002;542;21944;1873;20580;15218;4399;6700;2703;17057;17571
BIOCARTA_ETC_PATHWAY	7960;9578;6153;17494;1603;20586
BIOCARTA_NDKDYNAMIN_PATHWAY	21520;8473;1668;17216;20405;1670;1670;12421;13974
BIOCARTA_EPHA4_PATHWAY	16085;22110;18256;19236
BIOCARTA_EPO_PATHWAY	14573;10119;11352;21235;1873;4399;6700;2703;5023;17571
BIOCARTA_ECM_PATHWAY	10119;21235;18197;6847;1873;15218;2464;18256;9897
BIOCARTA_ERK_PATHWAY	10119;21235;16364;16568;20323;1873;6700;17571
BIOCARTA_ERYTH_PATHWAY	1524;20533;278;20431;11635;17955
BIOCARTA_EPONFKB_PATHWAY	5710;1842;15977;15570
BIOCARTA_EIF_PATHWAY	17218;10932;9145;3185;20765
BIOCARTA_EXTRINSIC_PATHWAY	6301;5440;18162;16057;17169
BIOCARTA_FAS_PATHWAY	14573;12094;11352;10477;2821;19948;16220;16931;19526;21944;21087;6225;12157;6410;6756;13150;882;9243;13188;14755
BIOCARTA_FCER1_PATHWAY	14573;10119;11352;8554;21235;1668;7231;14326;3307;4268;C10773.4;21944;1873;15293;11290;15218;4399;6700;5142;19236;17571
BIOCARTA_FEEDER_PATHWAY	1961;21646;16996;8301
BIOCARTA_FIBRINOLYSIS_PATHWAY	6301;8269;5440;2417;19476
BIOCARTA_FMLP_PATHWAY	10119;7115;8554;2700;21235;1842;1668;21944;8843;1873;21087;3699;5142;3992;15977;15570
BIOCARTA_FREE_PATHWAY	17458;823;1994;5108;8478;1842;13078;15977
BIOCARTA_GABA_PATHWAY	7284;20188;2758;3993;1987;8473;10305;18972
BIOCARTA_GATA3_PATHWAY	14967;11491;18959;21113;2928;7598;5142
BIOCARTA_GLYCOLYSIS_PATHWAY	8024;14852;12004;10159
BIOCARTA_SET_PATHWAY	5204;882;7957;15381;1670;16499;9489;14286
BIOCARTA_GH_PATHWAY	10119;21235;7993;10184;7762;20552;5023;1873;15218;10451;4399;6700;2703;16758;17571
BIOCARTA_AHSP_PATHWAY	12736;1174;20258;7397;12736;795;7686;9862
BIOCARTA_TCAPOPTOSIS_PATHWAY	3126;13150;21020;3817;12754;19241
BIOCARTA_HIVNEF_PATHWAY	10477;18255;13078;19708;22038;2821;16220;16931;19526;2013;18142;18485;11046;21944;11290;11310;6225;6410;18211;20580;6756;15977;15570;11 352;18197;1842;18118;19445;19948;20379;14008;14297;882;13150;9243;14755;22038
BIOCARTA_SALMONELLA_PATHWAY	16085;20055;4607;8266;10421
BIOCARTA_MPR_PATHWAY	2713;10119;16085;11491;4909;8005;21386;11809;18959;8266;11700;10421;2928;21982
BIOCARTA_HCMV_PATHWAY	21944;1842;1873;3699;20927;15218;15977
BIOCARTA_P53HYPOXIA_PATHWAY	11352;4307;12738;1334;11310;2490;9732;3210
BIOCARTA_HIF_PATHWAY	14573;319;10411;16430;9179
BIOCARTA_IGF1_PATHWAY	14573;10119;11352;21235;1873;20580;15218;16568;6700;17571
BIOCARTA_IL17_PATHWAY	8351;1494;2785;12754;19241;7135;278;21020;20431
BIOCARTA_IL2_PATHWAY	14573;10119;11352;21235;1873;21428;7231;6700;2703;15445;7387;17571
BIOCARTA_IL3_PATHWAY	10119;21235;1873;4226;6700;2703;5023;17571
BIOCARTA_IL4_PATHWAY	6700;7598;19306;17057;21428
BIOCARTA_IL5_PATHWAY	14820;8740;7598;9110;18322;20431
BIOCARTA_IL6_PATHWAY	14573;10119;21235;1873;6958;21428;9345;6700;20431;17571
BIOCARTA_IL10_PATHWAY	19536;14498;2002;13078;1220;1256;20533;17664;2703;17057;20431
BIOCARTA_IL12_PATHWAY	14573;17544;11352;13849;19536;5370;12754;19241;6958;3699;14330;3126;21020;12921
BIOCARTA_IL2RB_PATHWAY	10119;7852;21235;10184;19526;7231;5848;21263;15445;2138;5023;14297;21428;15218;13150;17977;6700;C12743;2703;17571
BIOCARTA_IL22BP_PATHWAY	2552;19536;14498;2002;6958;4035;21428;2703;21263;17057

BIOCARTA_IL7_PATHWAY	14297;7357;21428;15218;16499;C12743;2703;18256;22008;7387;6331
BIOCARTA_GSK3_PATHWAY	4925;18625;1842;14354;22473;8005;7832;18154;12264;15218;13783;15977
BIOCARTA_DEATH_PATHWAY	10477;18255;18118;1842;19708;5189;2821;19948;11118;16931;19526;16038;2013;20379;14008;188;14297;11046;6225;6410;882;15977;15570
BIOCARTA_RACCYCD_PATHWAY	10119;9028;7852;18255;21235;1842;11989;21087;15218;15977;21827;15570
BIOCARTA_GLEEVEC_PATHWAY	14573;10119;11352;21235;21944;14498;1873;15218;17977;6700;2703;5848;17571
BIOCARTA_INSULIN_PATHWAY	14573;10119;11352;21235;1873;7762;20580;15218;6700;16758;17571
BIOCARTA_INTEGRIN_PATHWAY	14573;10119;167;11352;16085;21235;10639;16994;9897;1873;2259;895;16271;17977;6700;18256;8192;17571;6978
BIOCARTA_INTRINSIC_PATHWAY	253;6301;15691;12660;21570;5440;13530;18162;7256;15688;14127;17169
BIOCARTA_KERATINOCYTE_PATHWAY	14573;10119;11352;18255;21235;7993;1842;13078;542;2013;20379;15669;18142;14674;18485;14449;14297;21944;13427;10577;1873;11290;3699;20927;6756;13150;14802;15977;15570
BIOCARTA_TCRA_PATHWAY	C12233.8;12754;19241;21020;18256;7387;7466
BIOCARTA_LECTIN_PATHWAY	16043;20822;1886;4429;14737
BIOCARTA_PYK2_PATHWAY	14573;10119;11352;21235;7993;21944;1873;2259;21087;17977;4399;6700;22008;17571
BIOCARTA_EGFR_SMRTE_PATHWAY	2380;21944;7227;1873;542
BIOCARTA_MAPK_PATHWAY	16911;14573;14031;18255;21235;14498;2013;18485;10090;1748;9739;3877;9217;21944;10577;1873;10344;11290;13522;3699;12157;13696;46;14930;6756;21517;6700;15070;15977;15570;7033;10119;11352;1842;13384;1924;20379;9572;14008;17955;21087;20927;414
BIOCARTA_MCALPAIN_PATHWAY	16186;10119;16085;10639;11491;542;2464;16994;9897;12921;18959;2928
BIOCARTA_PPARA_PATHWAY	14573;22436;7993;11491;13078;21853;14332;16499;2625;4240;2380;3711;156;19095;2703;15570;13120;21968;1842;14405;21589;14849;6034;18959;20927;18260;15218;2928;11752
BIOCARTA_ETS_PATHWAY	14573;9639;2380;10119;13571;21985;7852;19917;11453;10676;14802;12480
BIOCARTA_MONOCYTE_PATHWAY	4045;14437;14592;11361;18652
BIOCARTA_MTOR_PATHWAY	3804;15413;21963;17218;3185;22399;15218;20765;22252;5870;6794
BIOCARTA_IGF1R_PATHWAY	10119;21235;11491;1873;18959;15218;16568;6700;2928;3344;5848;17571
BIOCARTA_PITX2_PATHWAY	4925;6280;14354;12264;18260;16499;3390;12953;4785
BIOCARTA_NGF_PATHWAY	14573;10119;11352;21235;1873;15218;4399;6700;20323;17571
BIOCARTA_VIP_PATHWAY	9330;18255;11491;1842;1668;21944;18959;21956;4399;2928;9115;5142;15977;15570
BIOCARTA_NFAT_PATHWAY	16911;7115;21235;11491;1668;22250;16499;17807;1873;9179;3992;10119;11352;8554;16085;17723;14354;15504;4544;1710;18959;20181;15218;3969;13948;2928;446;5142;16998
BIOCARTA_NTHI_PATHWAY	9739;18255;1842;11190;13078;3699;12157;21589;16499;11741;20379;1114;9110;1378;15977;11499;9572;15570
BIOCARTA_NFKB_PATHWAY	9028;18255;21944;1842;13078;20533;12157;14252;16931;13783;2013;20379;15977;7832;15570;14008
BIOCARTA_NOS1_PATHWAY	17280;17438;9868;14551;7993;11491;20273;18959;1668;2928
BIOCARTA_NO2IL12_PATHWAY	17544;13849;19536;3711;2785;12754;6958;19241;14330;3126;21020;12921
BIOCARTA_RARRXR_PATHWAY	2380;15751;6189;18261;2415;17370;13968;2682
BIOCARTA_NUCLEARRS_PATHWAY	1785;5950;16366;2484;13916;11752;13039;20036;1532;2625
BIOCARTA_ARENRF2_PATHWAY	14573;11352;7993;13413;21023;8774;660;7019
BIOCARTA_P38MAPK_PATHWAY	16911;14031;10119;14498;13384;4756;2013;4048;9572;14008;17955;18485;21944;C10773.4;12157;3699;6756;6700
BIOCARTA_P53_PATHWAY	14297;7852;18118;12738;11989;11310;2490;13872;4196;21827
BIOCARTA_PDGF_PATHWAY	14573;10119;11352;19536;21235;14498;7993;2002;6034;21944;1873;20580;15218;4399;6700;2703;17057;17571
BIOCARTA_CCR5_PATHWAY	14573;11352;7993;5622;3126;4399;1469;22008
BIOCARTA_PTDINS_PATHWAY	4268;14354;10159;11187;20909;2144;4399;5848;102;19236;14674
BIOCARTA_PLCE_PATHWAY	17762;18837;2928;11491;18959;11855
BIOCARTA_EDG1_PATHWAY	7993;8005;7544;6034;18874;15218;3463;271;1405
BIOCARTA_CDK5_PATHWAY	8141;8699;10119;10484;20323;21235;1873
BIOCARTA_MYOSIN_PATHWAY	167;7993;21845;4183;6197;2464;16144;914;19009;10690;5569;6847;11187
BIOCARTA_PLATELETAPP_PATHWAY	253;15691;12660;21570;8269;15688;14127;2417
BIOCARTA_PS1_PATHWAY	4925;14354;14141;12264;20552;13587
BIOCARTA_PROTEASOME_PATHWAY	16498;20090;1293;10775;13893;12348;2792;3555;17685;17283
BIOCARTA_AKAPCENTROSOME_PATHWAY	20441;11491;18959;6857;4909;4369;2928;10690;5671;14674
BIOCARTA_PTEN_PATHWAY	3804;15413;2539;2259;15218;6700;17571
BIOCARTA_RAB_PATHWAY	9211;18164;16085;8786
BIOCARTA_RAC1_PATHWAY	8699;167;21944;8843;642;15293;4607;21087;1585;17243;15218;2464;7773;8141;19686
BIOCARTA_RAS_PATHWAY	10119;18255;21235;1842;1873;1585;15218;19686;5848;15977
BIOCARTA_NKCELLS_PATHWAY	8175;13913;5370;14441;1873;15293;21087;15218;3429;7231;22008;5023
BIOCARTA_RB_PATHWAY	2713;13123;11989;2068;4909;9099;11700;3344
BIOCARTA_CHREBP2_PATHWAY	19460;11491;13265;17542;9794;4000;4234;7021;10048;6491;19315;18959;17667;21175;4369;16481;3628;2928;9025;3468;14852;3344
BIOCARTA_BAD_PATHWAY	11491;4226;16568;278;5848;14297;12738;7618;18959;15218;2928;3344
BIOCARTA_CK1_PATHWAY	8699;4307;11491;18959;8141;2928;9718
BIOCARTA_EIF2_PATHWAY	3698;14354;12221;8797;7967
BIOCARTA_EIF4_PATHWAY	3804;15413;7993;3185;22399;15218;20765;5870
BIOCARTA_STEM_PATHWAY	1524;13571;7357;7598;20431;11635
BIOCARTA_P27_PATHWAY	7852;19893;11142;16153;21827
BIOCARTA_PGC1A_PATHWAY	16911;14031;4544;7115;194;2446;7762;1668;3344;3992;5480
BIOCARTA_PML_PATHWAY	10119;9839;13078;6756;13150;16499;2088
BIOCARTA_DREAM_PATHWAY	14573;11491;18959;2928
BIOCARTA_LEPTIN_PATHWAY	4369;6508;4234;10048;17667
BIOCARTA_RHO_PATHWAY	167;18197;2464;7773;7254;201;6847;15037;15653;8266;10421
BIOCARTA_AKAP13_PATHWAY	17264;4369;2928;11491;15214;18959;637
BIOCARTA_ATRBRCA_PATHWAY	14908;8521;12612;C11046.4;2656;13123;4933;8614;10860;15323
BIOCARTA_CARDIACEGF_PATHWAY	14573;10119;7993;1842;19742;542;9179;13948;4399;15977
BIOCARTA_HER2_PATHWAY	10119;21235;1873;15218;6700;20431;17571
BIOCARTA_ERK5_PATHWAY	16911;14031;10119;15218;4399;6700
BIOCARTA_MAL_PATHWAY	10119;11352;16085;21235;21944;1873;21119;20552;7773
BIOCARTA_MEF2D_PATHWAY	16186;14031;7993;10639;1668;3390;10676;5142;16994
BIOCARTA_MITOCHONDRIA_PATHWA	10477;14297;11046;6766;18118;12738;19708;6225;6410;5369;882;6561;18924;20935
BIOCARTA_ACH_PATHWAY	18084;2539;15986;15218;7197;3344;22008;5848
BIOCARTA_PARKIN_PATHWAY	9132;177;9671;9450;13501
BIOCARTA_CDC42RAC_PATHWAY	20055;21087;15218;8266;10421
BIOCARTA_RANMS_PATHWAY	3073;6325;6798;18309;8500
BIOCARTA_BARR_MAPK_PATHWAY	21235;1873;8473
BIOCARTA_TOB1_PATHWAY	3817;12754;19241;14330;21020;11741;1114;7598;20747;1378;15445;9572;17955
BIOCARTA_BARRESTIN_SRC_PATHWA	10119;21235;1873;8473
BIOCARTA_NKT_PATHWAY	1524;3817;13073;14330;19546;1469;17955;12921;12785;17544;13849;14820;21343;3126;22410;7598;19306
BIOCARTA_IL1R_PATHWAY	14573;11352;18255;1842;13078;14252;13343;20379;13784;9110;7832;17955;21944;12157;3699;20533;15977;20431;15570
BIOCARTA_MET_PATHWAY	14573;10119;11352;16085;21235;9897;3804;1873;21087;21301;20580;15218;16271;17977;6700;22008;17571
BIOCARTA_GPCR_PATHWAY	14573;10119;8554;21235;7993;11491;1668;8005;1873;18959;4399;2928;5142
BIOCARTA_IGF1MTOR_PATHWAY	3804;15413;14354;22399;15218;16568;5870;12221;6794
BIOCARTA_SODD_PATHWAY	16931;2013;13078;19708;14008
BIOCARTA_SHH_PATHWAY	20458;16863;14384;11491;14354;18959;4200;2928
BIOCARTA_PTC1_PATHWAY	9099;6040;11700;7820;16863;19910;9623;21982;11780;4909
BIOCARTA_SPRY_PATHWAY	10119;7219;21235;20900;1873;542;20580;6700;21983;17571
BIOCARTA_BARRESTIN_PATHWAY	8473
BIOCARTA_STATHMIN_PATHWAY	4544;194;2446;10577;11491;2785;12754;18959;19241;4909;21020;2928;21982
BIOCARTA_HSP27_PATHWAY	7033;14297;16085;18118;13078;13384;20533;6410;4756;6756;13150

BIOCARTA_TCR_PATHWAY	C12233.8;14573;10119;11352;8554;21235;7993;1842;1668;21020;21944;1873;15293;12754;19241;20580;15218;4399;6700;18256;5142;15977;7387;155
BIOCARTA_TCYTOTOXIC_PATHWAY	3817;2785;12754;19241;18652;358;21020;7466
BIOCARTA_THELPER_PATHWAY	3817;2785;12754;19241;18652;358;21020;7466
BIOCARTA_TALL1_PATHWAY	7380;11352;18255;1842;4149;14252;16981;10759;20379;1367;15977
BIOCARTA_TEL_PATHWAY	14297;18084;7993;4510;16568
BIOCARTA_TGFB_PATHWAY	1873;12157;18201;16499;11741;1114;8977;1378;9572;6940;5270;17955
BIOCARTA_TH1TH2_PATHWAY	12785;11671;17544;13849;20510;3817;5370;14330;19546;7598;19306;15445
BIOCARTA_41BB_PATHWAY	14573;18485;11352;18255;21944;1842;16755;46;14330;7598;15977;15570
BIOCARTA_KREB_PATHWAY	16309;3012;19365;21524
BIOCARTA_CTLA4_PATHWAY	20510;3817;12754;19241;6850;15218;12790;6700;21020;2871;7387;9038
BIOCARTA_LONGEVITY_PATHWAY	10119;823;5710;8603;2539;6298;15218;16568;10451
BIOCARTA_SARS_PATHWAY	14715;21182;20259
BIOCARTA_PAR1_PATHWAY	167;6301;7993;21845;4183;6197;8005;16144;914;19009;12157;15218;11187;22008
BIOCARTA_STRESS_PATHWAY	14573;9028;9400;11352;18255;1842;13078;2013;20379;14008;21944;3699;18211;15977;15570
BIOCARTA_TNFR1_PATHWAY	14573;11352;13078;19948;2821;16220;16931;2013;14008;21696;21944;21087;12157;6410;18211;882;9243;14755
BIOCARTA_TNFR2_PATHWAY	9028;9400;18255;14209;21944;1842;21589;10759;2013;20379;15977;15570
BIOCARTA_TOLL_PATHWAY	14573;9028;11352;6063;18255;18625;1842;7661;14252;4596;9520;20379;7832;21944;18154;12157;3699;13582;13783;15977;11499;15570
BIOCARTA_TPO_PATHWAY	14573;10119;21235;14498;7993;1873;12856;20580;15218;4399;6700;2703;17571
BIOCARTA_CREB_PATHWAY	10119;7993;11491;194;2446;18959;15218;6700;2928;17571
BIOCARTA_CARM1_PATHWAY	11491;18959;18261;16499;2928
BIOCARTA_TFF_PATHWAY	10119;18118;15218;10451;6700;5848;17571
BIOCARTA_TRKA_PATHWAY	15218;10119;4399;6700;20323;7993;17571
BIOCARTA_ARF_PATHWAY	7852;14574;21531;11310;15218;11728;20622
BIOCARTA_UCALPAIN_PATHWAY	16085;10639;19948;16994;9897;8192;6978
BIOCARTA_VEGF_PATHWAY	10119;7993;17589;12221;9897;319;6521;1195;10932;14748;15218;4399
BIOCARTA_VITCB_PATHWAY	15691;12660;21570;15688;10411;10825;14605
BIOCARTA_WNT_PATHWAY	4925;4307;14354;14141;16499;7528;1731;12157;12264;2484;3390;1114
BIOCARTA_ACTINY_PATHWAY	5875;20055;16085;2150;4607;8266;10421;8602;8055
