# Supplementary material for: Identifying Gene Set Association Enrichment Using the Coefficient of Intrinsic Dependence
Source: PLoS One. 2013 Mar 14;8(3):e58851. doi: 10.1371/journal.pone.0058851 (PMC3597597; doi:10.1371/journal.pone.0058851)
Supplement: Table S3 — GEO accession numbers of the samples used in the supervised GSAA. (PDF) [file pone.0058851.s004.pdf]

Table S3. GEO accession numbers of the samples used in the supervised GSAA.

Name of the subcohort	GEO accessions
LumA	GSM237141, GSM237151, GSM237153, GSM237156, GSM237157, GSM237158, GSM237165, GSM237168, GSM237174, GSM237175, GSM237176, GSM237177, GSM237179, GSM237183, GSM237184, GSM237185, GSM237191, GSM237198, GSM237199, GSM237200, GSM237203, GSM237250, GSM237267, GSM593757, GSM593759, GSM237139, GSM237143, GSM237149, GSM237155, GSM237180, GSM237182, GSM237195, GSM237197, GSM237248, GSM237266, GSM237274, GSM593782, GSM593783, GSM593784, GSM593756, GSM593762, GSM593763, GSM593691, GSM593692, GSM593693, GSM593695, GSM237162, GSM237163, GSM237164, GSM593690, GSM237172, GSM593696, GSM593697, GSM593699, GSM593700, GSM593701, GSM593702, GSM593704, GSM593709, GSM593705
LumB	GSM237137, GSM237150, GSM237154, GSM237167, GSM237194, GSM237278, GSM593750, GSM593751, GSM593752, GSM593753, GSM593754, GSM593785, GSM593773, GSM593765, GSM593788, GSM593790, GSM593791, GSM593792, GSM593755, GSM593796, GSM593797, GSM593798, GSM593801, GSM237140, GSM237181, GSM237186, GSM426480, GSM426481, GSM593749, GSM593795, GSM593691, GSM593692, GSM593693, GSM593695, GSM237162, GSM237163, GSM237164, GSM593690, GSM237172, GSM593696, GSM593697, GSM593699, GSM593700, GSM593701, GSM593702, GSM593704, GSM593709, GSM593705
152A	GSM593755, GSM593785, GSM593757, GSM237157, GSM237168, GSM237199, GSM593788, GSM593801, GSM237174, GSM593790, GSM593791, GSM237260, GSM237278, GSM593792, GSM237253, GSM237270, GSM237185, GSM237179, GSM237167, GSM237177, GSM593797, GSM237137, GSM237154, GSM237165, GSM237153, GSM593759, GSM237261, GSM237249, GSM237184, GSM593752, GSM593754, GSM237203, GSM237191, GSM593750, GSM593765, GSM237151, GSM237250, GSM237194, GSM237200, GSM593751, GSM237175, GSM237176, GSM593773, GSM593796, GSM593798, GSM593710, GSM237158, GSM237245, GSM593753, GSM237141, GSM237150, GSM237277, GSM593711, GSM237264, GSM237267, GSM237156, GSM237183, GSM237198, GSM237257, GSM237254, GSM237263, GSM237166, GSM237246, GSM593746, GSM593747, GSM593764, GSM593805, GSM237147, GSM237159, GSM237170, GSM237251, GSM593802, GSM237144, GSM237148, GSM237190, GSM237202, GSM237273, GSM237276, GSM593742, GSM593743, GSM593744, GSM593745, GSM593778, GSM593774, GSM593776, GSM593810, GSM593779, GSM593775, GSM593777, GSM593772, GSM593786, GSM593787, GSM593789, GSM593793, GSM593794, GSM593799, GSM593800, GSM593758, GSM593760, GSM593804, GSM593808, GSM237152, GSM237178, GSM237187, GSM237188, GSM237193, GSM237272, GSM593712, GSM593713, GSM593714, GSM593715, GSM593716, GSM593717, GSM593718, GSM593719, GSM593720, GSM593721, GSM593722, GSM593723, GSM593724, GSM593725, GSM593726, GSM593727, GSM593728, GSM593729, GSM593730, GSM593731, GSM593732, GSM593733, GSM593734, GSM593735, GSM593736, GSM593737, GSM593738, GSM593739, GSM593740, GSM593741, GSM593748, GSM593781, GSM593771,
NT	GSM593694, GSM237171, GSM593698, GSM593703, GSM593706, GSM593708, GSM593707, GSM593691, GSM593692, GSM593693, GSM593695, GSM237162, GSM237163, GSM237164, GSM593690, GSM237172, GSM593696, GSM593697, GSM593699, GSM593700, GSM593701, GSM593702, GSM593704, GSM593709, GSM593705
