# Supplementary material for: Identifying Gene Set Association Enrichment Using the Coefficient of Intrinsic Dependence
Source: PLoS One. 2013 Mar 14;8(3):e58851. doi: 10.1371/journal.pone.0058851 (PMC3597597; doi:10.1371/journal.pone.0058851)
Supplement: Table S4 — The genes in the signaling transduction pathways (STPs) of interest in the analysis of the supervised GSAA. (PDF) [file pone.0058851.s005.pdf]

Table S4. The genes in the signaling transduction pathways (STPs) of interest in the analysis of the supervised GSAA.

STPs	Agilent Feature Number
proteasomes	14330, 12061, 16498, 17283, 3555, 12664, 13893, 15417, 15593, 6249, 21436, 19568, 1293, 20090, 9949, 10775, 2792, 12348, 8278, 5162, 3797, 10706, 21010, 1422, 7546, 16413, 19577, 14704, 7269, 6493, 15647, 20521, 18690, 20474, 2880, 15618, 12025, 18765, 1815, 2845, 8132, 13938, 7124
PDGFRB	10898, 865, 1437, 1807, 1952, 2259, 2703, 3804, 3813, 3851, 4187, 4386, 4399, 4503, 4794, 5597, 5807, 6008, 6132, 6378, 6617, 6865, 6926, 7387, 7544, 8019, 8055, 8369, 8496, 9452, 9457, 10119, 10325, 10730, 10859, 11238, 11577, 12377, 13020, 13955, 14019, 14498, 14501, 14530, 14764, 14880, 15013, 15034, 15218, 15223, 16375, 16622, 16731, 17104, 17414, 17571, 17606, 18142, 19236, 19307, 19662, 19744, 19766, 20580, C11176.3
