# Supplementary material for: Identifying Gene Set Association Enrichment Using the Coefficient of Intrinsic Dependence
Source: PLoS One. 2013 Mar 14;8(3):e58851. doi: 10.1371/journal.pone.0058851 (PMC3597597; doi:10.1371/journal.pone.0058851)
Supplement: Table S5 — Significant associated pathways in KEGG database using 25 tumor samples (25T) and 25 nontumor samples (25N). The rows are predictors and the columns are the targets. '11' (yellow) denotes 18 significant associations in both 25T and 25N. '10' (red) denotes 380 significant associations in 25A but not in 25N. '1' (green) denotes 2724 significant associations in 25N but not in 25T. (PDF) [file pone.0058851.s006.pdf]

Table S5. Significant associated pathways in KEGG database using 25 tumor samples (25T) and 25 nontumor samples (25N). The rows are predictors and the columns are the targets. '11' (yellow) denotes 18 significant associations in both 25T and 25N. '10' (red) denotes 380 significant associations in 25A but not in 25N. '1' (green) denotes 2724 significant associations in 25N but not in 25T.

GLYCOLYSIS_GLUCONEOGENESIS	[illegible]
CITRATE_CYCLE_TCA_CYCLE	[illegible]
PENTOSE_PHOSPHATE_PATHWAY	[illegible]
PENTOSE_AND_GLUCURONATE_INTERCONVERSIONS	[illegible]
FRUCTOSE_AND_MANNOSE_METABOLISM	[illegible]
GALACTOSE_METABOLISM	[illegible]
ASCORBATE_AND_ALDARATE_METABOLISM	[illegible]
FATTY_ACID_METABOLISM	[illegible]
STEROID_BIOSYNTHESIS	[illegible]
PRIMARY_BILE_ACID_BIOSYNTHESIS	[illegible]
STEROID_HORMONE_BIOSYNTHESIS	[illegible]
OXIDATIVE_PHOSPHORYLATION	[illegible]
PURINE_METABOLISM	[illegible]
PYRIMIDINE_METABOLISM	[illegible]
ALANINE_ASPARTATE_AND_GLUTAMATE_METABOLISM	[illegible]
GLYCINE_SERINE_AND_THREONINE_METABOLISM	[illegible]
CYSTEINE_AND_METHIONINE_METABOLISM	[illegible]
VALINE_LEUCINE_AND_ISOLEUCINE_DEGRADATION	[illegible]
VALINE_LEUCINE_AND_ISOLEUCINE_BIOSYNTHESIS	[illegible]
LYSINE_DEGRADATION	[illegible]
ARGININE_AND_PROLINE_METABOLISM	[illegible]
HISTIDINE_METABOLISM	[illegible]
TYROSINE_METABOLISM	[illegible]
PHENYLALANINE_METABOLISM	[illegible]
TRYPTOPHAN_METABOLISM	[illegible]
BETA_ALANINE_METABOLISM	[illegible]
TAURINE_AND_HYPOTAURINE_METABOLISM	[illegible]
SELENOAMINO_ACID_METABOLISM	[illegible]
GLUTATHIONE_METABOLISM	[illegible]
STARCH_AND_SUCROSE_METABOLISM	[illegible]
N_GLYCAN_BIOSYNTHESIS	[illegible]
OTHER_GLYCAN_DEGRADATION	[illegible]
O_GLYCAN_BIOSYNTHESIS	[illegible]
AMINO_SUGAR_AND_NUCLEOTIDE_SUGAR_METABOLISM	[illegible]
GLYCOSAMINOGLYCAN_DEGRADATION	[illegible]
GLYCOSAMINOGLYCAN_BIOSYNTHESIS_CHONDROITIN_SULFATE	[illegible]
GLYCOSAMINOGLYCAN_BIOSYNTHESIS_KERATAN_SULFATE	[illegible]
GLYCOSAMINOGLYCAN_BIOSYNTHESIS_HEPARAN_SULFATE	[illegible]
GLYCEROLIPID_METABOLISM	[illegible]
INOSITOL_PHOSPHATE_METABOLISM	[illegible]
GLYCOSYLPHOSPHATIDYLINOSITOL_GPI_ANCHOR_BIOSYNTHESIS	[illegible]
GLYCEROPHOSPHOLIPID_METABOLISM	[illegible]
ETHER_LIPID_METABOLISM	[illegible]
ARACHIDONIC_ACID_METABOLISM	[illegible]
LINOLEIC_ACID_METABOLISM	[illegible]
ALPHA_LINOLENIC_ACID_METABOLISM	[illegible]
SPHINGOLIPID_METABOLISM	[illegible]
GLYCOSPHINGOLIPID_BIOSYNTHESIS_LACTO_AND_NEOLACTO_SERIES	[illegible]
GLYCOSPHINGOLIPID_BIOSYNTHESIS_GLOBO_SERIES	[illegible]
GLYCOSPHINGOLIPID_BIOSYNTHESIS_GANGLIO_SERIES	[illegible]
PYRUVATE_METABOLISM	[illegible]
GLYOXYLATE_AND_DICARBOXYLATE_METABOLISM	[illegible]
PROPANOATE_METABOLISM	[illegible]
BUTANOATE_METABOLISM	[illegible]
ONE_CARBON_POOL_BY_FOLATE	[illegible]
RIBOFLAVIN_METABOLISM	[illegible]
NICOTINATE_AND_NICOTINAMIDE_METABOLISM	[illegible]
PANTOTHENATE_AND_COA_BIOSYNTHESIS	[illegible]
FOLATE_BIOSYNTHESIS	[illegible]
RETINOL_METABOLISM	[illegible]
PORPHYRIN_AND_CHLOROPHYLL_METABOLISM	[illegible]
TERPENOID_BACKBONE_BIOSYNTHESIS	[illegible]
LIMONENE_AND_PINENE_DEGRADATION	[illegible]
NITROGEN_METABOLISM	[illegible]
SULFUR_METABOLISM	[illegible]
AMINOACYL_TRNA_BIOSYNTHESIS	[illegible]
METABOLISM_OF_XENOBIOTICS_BY_CYTOCHROME_P450	[illegible]
DRUG_METABOLISM_CYTOCHROME_P450	[illegible]
DRUG_METABOLISM_OTHER_ENZYMES	[illegible]
BIOSYNTHESIS_OF_UNSATURATED_FATTY_ACIDS	[illegible]
ABC_TRANSPORTERS	[illegible]
RIBOSOME	[illegible]
RNA_DEGRADATION	[illegible]
RNA_POLYMERASE	[illegible]
BASAL_TRANSCRIPTION_FACTORS	[illegible]
DNA_REPLICATION	[illegible]
SPLICEOSOME	[illegible]
PROTEASOME	[illegible]
PROTEIN_EXPORT	[illegible]
PPAR_SIGNALING_PATHWAY	[illegible]
BASE_EXCISION_REPAIR	[illegible]
NUCLEOTIDE_EXCISION_REPAIR	[illegible]
MISMATCH_REPAIR	[illegible]
HOMOLOGOUS_RECOMBINATION	[illegible]
NON_HOMOLOGOUS_END_JOINING	[illegible]
MAPK_SIGNALING_PATHWAY	[illegible]
ERBB_SIGNALING_PATHWAY	[illegible]
CALCIUM_SIGNALING_PATHWAY	[illegible]
CYTOKINE_CYTOKINE_RECEPTOR_INTERACTION	[illegible]
CHEMOKINE_SIGNALING_PATHWAY	[illegible]
PHOSPHATIDYLINOSITOL_SIGNALING_SYSTEM	[illegible]

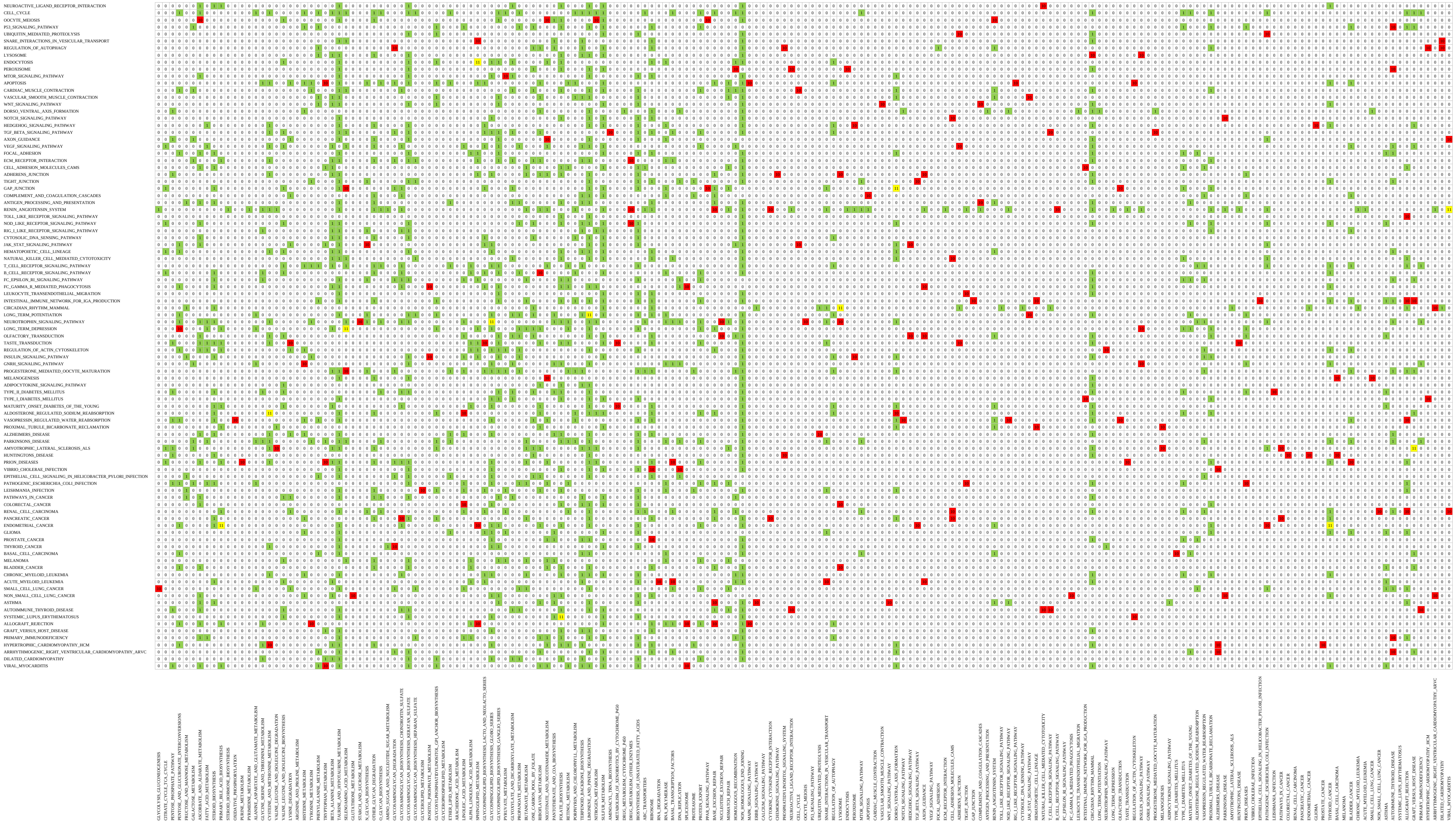
NEUROACTIVE_LIGAND_RECEPTOR_INTERACTION
CELL_CYCLE
OOCYTE_MEIOSIS
P53_SIGNALING_PATHWAY
UBIQUITIN_MEDIATED_PROTEOLYSIS
SNARE_INTERACTIONS_IN_VESICULAR_TRANSPORT
REGULATION_OF_AUTOPHAGY
LYSOSOME
ENDOCYTOSIS
PEROXISOME
MTOR_SIGNALING_PATHWAY
APOPTOSIS
CARDIAC_MUSCLE_CONTRACTION
VASCULAR_SMOOTH_MUSCLE_CONTRACTION
WNT_SIGNALING_PATHWAY
DORSO_VENTRAL_AXIS_FORMATION
NOTCH_SIGNALING_PATHWAY
HEDGEHOG_SIGNALING_PATHWAY
TGF_BETA_SIGNALING_PATHWAY
AXON_GUIDANCE
VEGF_SIGNALING_PATHWAY
FOCAL_ADHESION
ECM_RECEPTOR_INTERACTION
CELL_ADHESION_MOLECULES_CAMS
ADHERENS_JUNCTION
TIGHT_JUNCTION
GAP_JUNCTION
COMPLEMENT_AND_COAGULATION_CASCADES
ANTIGEN_PROCESSING_AND_PRESENTATION
RENIN_ANGIOTENSIN_SYSTEM
TOLL_LIKE_RECEPTOR_SIGNALING_PATHWAY
NOD_LIKE_RECEPTOR_SIGNALING_PATHWAY
RIG_I_LIKE_RECEPTOR_SIGNALING_PATHWAY
CYTOSOLIC_DNA_SENSING_PATHWAY
JAK_STAT_SIGNALING_PATHWAY
HEMATOPOIETIC_CELL_LINEAGE
NATURAL_KILLER_CELL_MEDIATED_CYTOTOXICITY
T_CELL_RECEPTOR_SIGNALING_PATHWAY
B_CELL_RECEPTOR_SIGNALING_PATHWAY
FC_EPSILON_RI_SIGNALING_PATHWAY
FC_GAMMA_R_MEDIATED_PHAGOCYTOSIS
LEUKOCYTE_TRANSENDOTHELIAL_MIGRATION
INTESTINAL_IMMUNE_NETWORK_FOR_IGA_PRODUCTION
CIRCADIAN_RHYTHM_MAMMAL
LONG_TERM_POTENTIATION
NEUROTROPHIN_SIGNALING_PATHWAY
LONG_TERM_DEPRESSION
OLFACTORY_TRANSDUCTION
TASTE_TRANSDUCTION
REGULATION_OF_ACTIN_CYTOSKELETON
INSULIN_SIGNALING_PATHWAY
GNRH_SIGNALING_PATHWAY
PROGESTERONE_MEDIATED_OOCYTE_MATURATION
MELANOGENESIS
ADIPOCYTOKINE_SIGNALING_PATHWAY
TYPE_II_DIABETES_MELLITUS
TYPE_I_DIABETES_MELLITUS
MATURITY_ONSET_DIABETES_OF_THE_YOUNG
ALDOSTERONE_REGULATED_SODIUM_REABSORPTION
VASOPRESSIN_REGULATED_WATER_REABSORPTION
PROXIMAL_TUBULE_BICARBONATE_RECLAMATION
ALZHEIMERS_DISEASE
PARKINSONS_DISEASE
AMYOTROPHIC_LATERAL_SCLEROSIS_ALS
HUNTINGTONS_DISEASE
PRION_DISEASES
VIBRIO_CHOLERAE_INFECTION
EPITHELIAL_CELL_SIGNALING_IN_HELICOBACTER_PYLORI_INFECTION
PATHOGENIC_ESCHERICHIA_COLI_INFECTION
LEISHMANIA_INFECTION
PATHWAYS_IN_CANCER
COLORECTAL_CANCER
RENAL_CELL_CARCINOMA
PANCREATIC_CANCER
ENDOMETRIAL_CANCER
GLIOMA
PROSTATE_CANCER
THYROID_CANCER
BASAL_CELL_CARCINOMA
MELANOMA
BLADDER_CANCER
CHRONIC_MYELOID_LEUKEMIA
ACUTE_MYELOID_LEUKEMIA
SMALL_CELL_LUNG_CANCER
NON_SMALL_CELL_LUNG_CANCER
ASTHMA
AUTOIMMUNE_THYROID_DISEASE
SYSTEMIC_LUPUS_ERYTHEMATOSUS
ALLOGRAFT_REJECTION
GRAFT_VERSUS_HOST_DISEASE
PRIMARY_IMMUNODEFICIENCY
HYPERTROPHIC_CARDIOMYOPATHY_HCM
ARRHYTHMOGENIC_RIGHT_VENTRICULAR_CARDIOMYOPATHY_ARVC
DILATED_CARDIOMYOPATHY
VIRAL_MYOCARDITIS
