# Supplementary figures and images for: Identifying Gene Set Association Enrichment Using the Coefficient of Intrinsic Dependence
Source: PLoS One. 2013 Mar 14;8(3):e58851. doi: 10.1371/journal.pone.0058851 (PMC3597597; doi:10.1371/journal.pone.0058851)

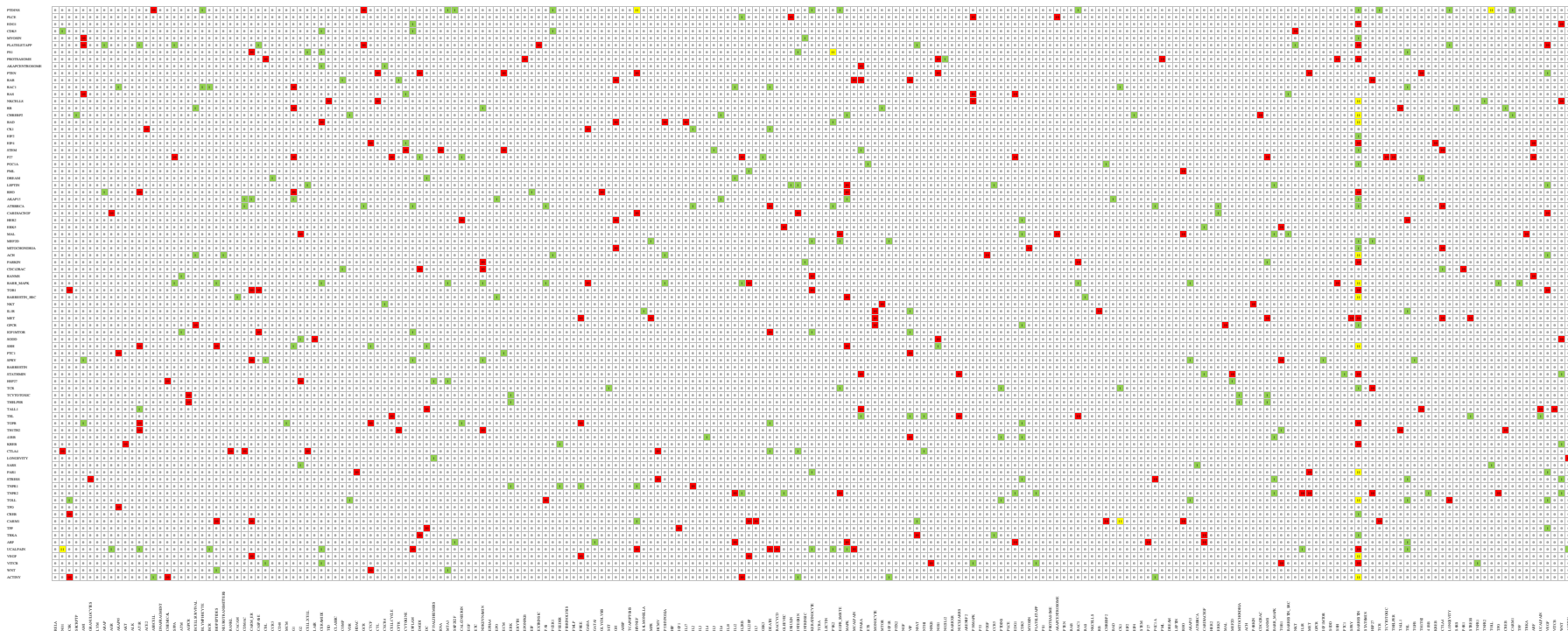

Supplement: Table S6 — Significant associated pathways in BioCarta database using 25 tumor samples (25T) and 25 nontumor samples (25N). The rows are predictors and the columns are the targets. '11' (yellow) denotes 34 significant associations in both 25T and 25N. '10' (red) denotes 692 significant associations in 25A but not in 25N. '1' (green) denotes 693 significant associations in 25N but not in 25T. (PDF) [file pone.0058851.s007.pdf]
